# Participatory approaches to climate adaptation, resilience, and mitigation: A systematic review

**DOI:** 10.1007/s13280-025-02202-z

**Published:** 2025-06-04

**Authors:** Meg Parsons, Naomi Joy Godden, Karen Paiva Henrique, Petra Tschakert, Noémi Gonda, Ed Atkins, Karin Steen, Roa Petra Crease

**Affiliations:** 1 School of Environment, The University of Auckland, Auckland, 1142 New Zealand; 2 Centre for People, Place and Planet and School of Arts and Humanities, Edith Cowan University, Joondalup, Australia; 3 Department of Human Geography, Planning and International Development, University of Amsterdam, Amsterdam, Netherlands; 4 Faculty of Humanities, School of Media, Creative Arts and Social Inquiry, Curtin University, Bentley, WA Australia; 5 Department of Urban and Rural Development, Swedish University of Agricultural Sciences, Uppsala, Sweden; 6 School of Geographical Sciences, University of Bristol, Bristol, England; 7 Lund University Centre for Sustainability Studies, Lund University, Lund, Sweden; 8 School of Environment, The University of Auckland, Auckland, New Zealand

**Keywords:** Climate adaptation, Climate mitigation, Empowerment, Participation, Power dynamics, Resilience

## Abstract

For decades, social scientists and activists have advocated for equal participation of citizens in decision-making about development and equitable futures. The urgency of this call has intensified with the climate emergency, pushing communities towards resilience. Yet, the concept of participation remains contested. This systematic review examines 187 articles to understand how participatory processes in climate mitigation, adaptation, and resilience initiatives engage with power dynamics. Findings are categorised into three themes: domination and exclusion, negotiation and contestation, and transformation and emancipation. Results reveal that most participatory approaches sustain power imbalances and exclude marginalised groups, hindering meaningful engagement. However, some cases highlight transformative practices that foster empowerment, shared decision-making, and inclusivity. These examples offer pathways to rethink participatory climate action by emphasising power redistribution and community leadership. Future climate initiatives must prioritise these transformative dimensions to disrupt entrenched inequalities and achieve just and resilient outcomes.

**Supplementary Information:**

The online version contains supplementary material available at 10.1007/s13280-025-02202-z.

## Introduction

As the climate emergency escalates, there is an urgent need for transformative climate action that prioritises meaningful public participation. Participation is widely advocated by scholars as essential for equitable climate governance, mitigation, and adaptation (Davies and Hügel 2021) and is enshrined within international climate agreements (Paris Agreement, see Horowitz 2016). However, meaningful participation remains contested and under-realised despite long-standing advocacy for equitable citizen involvement (Mees et al. 2019). Critics highlight the significant gap between participation rhetoric and reality, noting that many processes fail to redistribute power or challenge entrenched inequalities, often reinforcing tokenism and exclusion and, more recently, climate mitigation and adaptation (Araos 2023), participation remains a highly contested and under-realised ideal (Cooke and Kothari 2001). Critics highlight the significant gap between participation rhetoric and reality, noting that many processes fail to redistribute power or challenge entrenched inequalities, often reinforcing tokenism and exclusion (Cooke and Kothari 2001).

This paper conceptualises emancipatory participation as an engagement approach that actively challenges structural inequalities within climate mitigation, adaptation, and resilience processes. Unlike instrumental participation, which reinforces existing power hierarchies (Mason and Niewolny 2022), or deliberative participation, which prioritises consensus over systemic change, emancipatory participation seeks to dismantle oppressive structures (Devaney et al. 2020). Drawing on Freire’s (1986) pedagogy, we argue participation must actively contest dominant power relations, aligning with climate justice scholarship advocating redistributive, procedural, and recognitional justice (Holland 2017).

Recognising uneven Global North and Global South power dynamics, this paper critiques Eurocentric participatory frameworks that overlook diverse contexts and local realities (Sultana 2022). We investigate how participation operates differently across political contexts, interrogating its role in reproducing or challenging existing power asymmetries.

Participatory approaches to climate mitigation, adaptation, and governance are shaped by profoundly uneven power dynamics between the Global North and Global South. While international climate agreements and national adaptation plans promote participation as a universal good, the ways participation is conceptualised and operationalised often reflect Eurocentric governance norms, overlooking the diverse political, historical, and cultural contexts that shape local realities (Nagoda and Nightingale 2017). Instead, this paper examines how participation functions across different political and governance landscapes, critically interrogating how participatory processes reproduce or challenge existing power asymmetries in Global North and Global South contexts.

We systematically review 187 peer-reviewed publications (2015–2020), guided by the research question: How do participatory practices in climate mitigation and adaptation reinforce or challenge power structures, and what transformative potential do they hold? Our review identifies a continuum of participation: (1) domination and exclusion, (2) negotiation and contestation, and (3) transformation and emancipation. Our analysis emphasises that genuinely emancipatory participation must involve deliberate power shifts, embrace conflict as productive, and amplify marginalised voices. Ultimately, we argue for reframing participation from procedural engagement to a liberatory practice, aligned with decolonising environmental governance and fostering transformative resilience.

Our paper takes stock of participation's role in driving transformational change to address rising greenhouse gas emissions, worsening climate risks, and enhancing climate resilience. We argue that for participation to be genuinely emancipatory, it must be grounded in deliberate power shifts, embrace conflict as a productive force, and create spaces where marginalised voices can lead. In doing so, we aim to chart a path forward for participatory climate governance that is both just and transformative, capable of addressing the deep-rooted inequities that fuel climate vulnerability. This reframing of participation addresses the immediate challenges of climate adaptation and aligns with broader movements towards decolonising environmental governance and fostering resilience. By shifting the focus from participation as a procedural tool to participation as a practice of liberation, we highlight the transformative potential of climate mitigation and adaptation processes that are genuinely participatory, inclusive, and just.

The paper proceeds as follows: First, we overview conceptualisations of participation and power dynamics; second, we detail our systematic review methodology; third, we present our findings according to the identified continuum; and finally, we discuss implications and offer recommendations for inclusive, transformative climate participation.

## Participation

Participation is conceptualised diversely, reflecting disciplinary, theoretical, and contextual variations (Ianniello et al. 2019). Arnstein’s (1969) ladder remains influential (Gaber 2019), positioning participation from manipulation to citizen control. Arnstein emphasises power dynamics, arguing genuine participation requires transferring control from decision-makers to marginalised populations. For Arnstein, participation is about power dynamics and structures, with genuine participation requiring the transfer of control from decision-makers (i.e. international funders and government officials) to local actors and marginalised populations to allow them to shape the decisions that affect their lives (Arnstein 1969). Both drawing on and critiquing Arnstein’s work, many scholars frame participation as a struggle for power between different actors and institutions (Botchwey et al. 2019). Davidson (1998) proposes a dynamic alternative—the wheel of participation—which highlights participation’s cyclical nature across information, consultation, participation, and empowerment modes shaped by institutional constraints and social contexts.

However, scholars critique conventional participatory processes for frequently reinforcing existing inequalities and serving dominant power structures (Ranganathan and Bratman 2021). Particularly within climate adaptation and development contexts, participatory methods often remain symbolic rather than transformative (Eriksen et al. 2021). Indigenous (Chilisa 2019) and Global South scholars (Escobar 2015) critique externally imposed participation models for ignoring local Indigenous and local knowledge systems. Escobar (2011) and Indigenous scholars argue that conventional participation perpetuates colonial power structures, advocating instead for Indigenous-centred participatory frameworks that emphasise kinship, intergenerational equity, and more-than-human relationships (Smith 1999; Coombes 2017; McGregor 2018).

Non-Indigenous scholars similarly critique conventional participation, highlighting its neglect of collective action and more-than-human relations (Tschakert et al. 2021). Chilvers and Kearns (2020) propose a relational model emphasising reciprocal interactions among people, technologies, and environments. Hügel and Davies (2020) suggest that genuinely transformative participation requires deliberative dialogue and shared decision-making to ensure equitable outcomes. These perspectives underscore the significance of power dynamics, context, and equity in shaping participatory processes.

Drawing on the foundational works and contemporary critiques of participatory approaches (Davidson 1998; Hügel and Davies 2020), this paper reimagines participation's role in climate action. We argue conventional approaches—primarily consultative and superficial—reinforce existing power inequalities. To address these limitations, we advocate for transformative participation (Bladt and Percy-Smith 2021), deliberately challenging existing power structures to achieve equitable climate governance; this aligns with broader calls for transformative adaptation, intersectional climate justice, and decolonising frameworks (Porter et al. 2020), which seek to dismantle the colonial, racist, patriarchal, and classist formations that continue to shape climate governance, mitigation, and adaptation responses.

Our systematic review deliberately integrates adaptation, mitigation, and resilience as distinct but interconnected dimensions of climate governance and actions to address climate change. While these terms are often used interchangeably in policy discourse, they refer to different governance mechanisms and strategies. Adaptation encompasses adjustments to social and ecological systems in response to climate risks, mitigation focuses on reducing greenhouse gas emissions and enhancing carbon sinks, and resilience emphasises the capacity of systems to absorb and recover from shocks and disruptions. Each domain involves participatory approaches, but their implementation may vary due to differences in governance scales, funding priorities, and institutional frameworks.

The inclusion of all three dimensions reflects contemporary global climate governance frameworks, such as the Paris Agreement, the Sustainable Development Goals, and IPCC special and assessment reports, which highlight the growing imperative for participatory decision-making across climate actions. Many climate initiatives—particularly in the Global South—integrate adaptation, mitigation, and resilience within a single project. For instance, community-led reforestation programmes may be framed as a mitigation strategy (carbon sequestration), an adaptation intervention (reducing extreme heat events), and a resilience-building effort (enhancing biodiversity and local livelihoods) (Locatelli et al. 2011).

## Materials and methods

Our study employed a systematic review methodology (Tschakert et al. 2023) to critically evaluate how participation has been conceptualised and practised within climate resilience scholarship from 2015 to 2020. Systematic reviews are essential to comprehensively assess and synthesise existing literature, providing insights into overarching trends and identifying knowledge gaps (Berrang-Ford et al. 2021; Sharifi et al. 2021). We used clearly defined inclusion and exclusion criteria based on thematic relevance, geographical scope, and disciplinary focus (see Online Resource: Detailed Methodology for detailed criteria and rationale). Our review sought peer-reviewed journal articles that explicitly addressed participation in climate resilience contexts, utilising thematic content analysis to distil and interpret patterns from the selected texts.

Our search initially yielded 1247 journal articles from the academic databases Scopus guided by a comprehensive keyword strategy (detailed fully Online Resource Figure S1 and Online Resource Figure S2). Following removal of duplicates and screening of abstracts and full texts against our inclusion criteria, a total of 187 articles were selected for detailed review (see Online Resource Table S2, which provides the full references to all articles reviewed in this paper). We meticulously coded these articles based on thematic relevance, type of participation, power dynamics, and the geographic context of each study (Braun et al. 2022). This coding framework facilitated our analysis, enabling us to systematically categorise each article according to emerging themes and participation typologies.

The analysis phase involved an iterative thematic approach informed by constructivist grounded theory, allowing themes to evolve from the data itself rather than strictly adhering to predetermined categories (Johnson 2014). Each article was read multiple times by at least two researchers to ensure rigour and consistency in coding, with discrepancies resolved through consensus discussions. Throughout our review, we paid careful attention to positionality and reflexivity, explicitly acknowledging the ways in which our own academic and disciplinary backgrounds could influence interpretation (Fisher 2014; Braun and Clarke 2019). To further enhance methodological transparency and rigour, we conducted regular meetings to reflect collectively on emerging findings, coding choices, and interpretive decisions (Saldana 2016).

Finally, to validate our thematic findings, preliminary results were presented to a small expert panel consisting of scholars specialised in climate resilience, participation, and power dynamics. Feedback from this group was used to refine our interpretations and confirm the robustness and applicability of the key thematic categories identified. Methodological limitations and additional reflections on our analytical approach are detailed in Online Resource (Detailed Methodology), explicitly addressing potential biases and areas for future methodological improvement. Our systematic review approach thus enabled a comprehensive and critically reflective analysis of participation within climate resilience literature, highlighting both strengths and critical gaps in current scholarly approaches.

A systematic literature review approach was selected to critically examine overarching themes and patterns in climate mitigation, adaptation, and resilience literature, minimising biases associated with traditional narrative reviews. This approach is valuable for synthesising extensive, interdisciplinary research across diverse geographic and socio-political contexts, which aligns with the complex nature of participatory climate mitigation, adaptation, and resilience efforts (Nikravech et al. 2020; Owen 2020). Our review aimed to explore how participatory practices in climate actions reinforce or challenge power structures, focusing specifically on articles published between 2015 and 2020 to capture contemporary scholarship post-Paris Agreement (Belfer et al. 2019; Thew et al. 2021).

### Positionality and acknowledging the methodological limitations

Despite the comprehensive nature of our review, several limitations remain. Our reliance on peer-reviewed, English-language articles from Scopus excludes valuable non-academic sources and non-English contributions, particularly those capturing grassroots and Indigenous and local knowledge systems. Furthermore, keyword selection inevitably shapes the review’s scope, meaning alternate terms like "community-based adaptation", "citizen science", or "co-production" might yield additional relevant studies. Future research should incorporate grey literature, non-English publications, and broader keyword searches to enhance inclusivity.

We also acknowledge the positionality of our predominantly Global North-based research team, which inevitably influences methodological decisions and interpretations. Although the coding and author teams include members from Global North (Australia, New Zealand, Sweden, the UK, and the USA) and South (Brazil, Ghana, the Philippines, and South Africa), most remain within academic contexts shaped by Global North conventions. We encourage future research to integrate diverse methodological practices, field-based participatory methods, and greater collaboration with Global South and Indigenous scholars to comprehensively understand participation dynamics in climate mitigation, adaptation, and resilience.

## Results

**Fig. 1:**
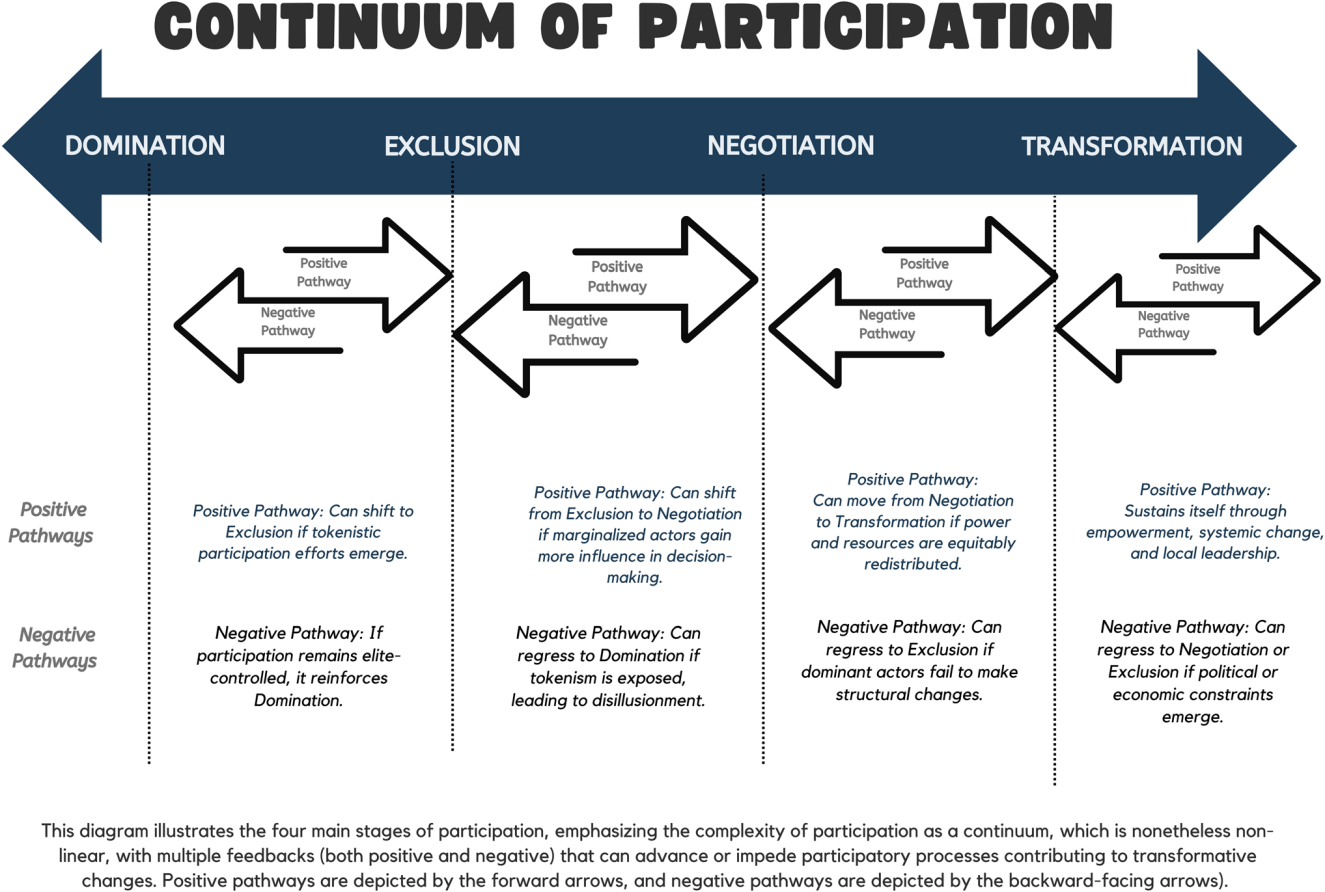
Continnum of participation

Our systematic review identified clear patterns of participation across the analysed literature. In this section, we first provide an overview of the geographical, institutional, and thematic dimensions of the articles reviewed before exploring the three distinct types of participation that emerged: participation as domination and exclusion (4.1), negotiation and contestation (4.2), and transformation and emancipation (4.3). These categories reveal participation as operating along a continuum rather than within discrete categories (Fig. 1). In our view, this continuum approach better reflects the relational, context-specific, and dynamic nature of participation compared to traditional static frameworks such as Arnstein's Ladder.

A notable finding was the geographical unevenness within the literature reviewed. Approximately 62% of the studies focused on Global South contexts, whereas 38% examined situations in the Global North. Despite the geographic diversity of case studies, authors predominantly held affiliations with institutions located in the Global North, while researchers based within Global South contexts were underrepresented (see Online Resource Table S2 for full references to Articles 52, 84, 130, 156, 161). This disparity signals persistent epistemological biases in the field, whereby conceptual frameworks and models generated in Global North institutions are uncritically deployed within Global South contexts, often without adequate consideration of local socio-political structures, cultural contexts, or governance systems.

Additionally, we observed distinct differences in the participatory approaches between climate mitigation, adaptation, and resilience-building projects. Mitigation projects, characterised by initiatives such as renewable energy developments, carbon offsetting, and REDD + schemes, predominantly featured top-down governance and technocratic decision-making processes that typically excluded or minimised local community participation (Articles 24, 72, 105, 119). Conversely, adaptation and resilience-building initiatives more commonly employed multi-scalar governance strategies and community engagement mechanisms but nonetheless often remained constrained by existing institutional hierarchies, donor expectations, and structural inequalities (Articles 7, 18, 35, 40, 91).

### Participation as domination and exclusion

Participation as domination and exclusion emerged most frequently within our reviewed sample, identified clearly in approximately 35% of the articles. Domination in participatory processes took diverse forms but consistently reflected hierarchical control by government agencies, international NGOs, elite stakeholders, and technical experts. Government-led participatory processes were prominent across all contexts, significantly shaping decision-making spaces and limiting meaningful involvement by local and marginalised communities (Articles 7, 35, 40, 91). Elite-driven processes similarly maintained exclusionary practices, privileging the priorities and interests of affluent stakeholders over those of disadvantaged or marginalised groups (Articles 47, 68, 91).

The manifestation of domination and exclusion was notably context-specific, with clear distinctions between Global South and Global North case studies. In Global South contexts, participation frequently involved externally imposed climate agendas—such as large-scale renewable energy projects and international REDD + schemes—that prioritised international donor interests, corporate profit, and government control over local community needs and rights (Articles 83, 119, 146). Such top-down initiatives generally engaged local communities superficially, conducting minimal consultation and offering limited or no opportunities for meaningful input or decision-making. These projects often reinforced existing inequalities and overlooked local land-use practices, governance systems, and Indigenous and Local knowledge (ILK) systems (Articles 69, 119, 150).

In contrast, domination in Global North contexts typically involved the use of deliberative participatory frameworks which presented as inclusive yet continued to privilege technical expertise and elite stakeholders' interests. These ostensibly participatory forums frequently marginalised low-income communities, racial minorities, and Indigenous peoples by limiting participation to consultative rather than decision-making roles (Articles 88, 105, 151). For example, in urban resilience projects and municipal climate adaptation planning in North America and Europe, technocratic planning and expert-driven strategies routinely marginalised affected communities' lived experiences and socio-economic realities, further perpetuating exclusion (Articles 88, 106, 135).

Domination emerged in various dimensions within our reviewed studies. Firstly, domination repeatedly materialised through top-down, government-, expert-, or elite-driven processes, often excluding marginalised groups from meaningful participation (Articles 7, 35, 40, 91, 128, 131, 144, 147, 156, 157, 161, 187). Within our sample, top-down exclusionary processes were more prevalent in the Global South (Articles 38, 48, 70, 104, 123, 125, 154, 166, 170, 171) compared to the Global North (Articles 38, 48, 60). Government officials constituted the most common stakeholder group, whereas marginalised populations such as women, people with disabilities, youth, ethnic minorities, and Indigenous peoples, including their knowledges and experiences, were frequently excluded (Articles 7, 147, 161). Consequently, many governmental and NGO initiatives lacked accurate insights into local conditions and priorities (Article 31).

Secondly, domination manifested in the disproportionate influence of social elites who not only participated more frequently but exerted greater control within decision-making spaces compared to marginalised groups (Articles 90, 95, 121, 151). In the Global South, local elites—such as village chiefs, elders, and politicians—often monopolised decision-making, deliberately restricting broader community participation (Articles 90, 95). Similarly, in the Global North, evidence showed that adaptation planning processes were often aligned with elite preferences, neglecting vulnerable populations' needs (Articles 88, 121, 151). For instance, local government-led adaptation planning in Virginia, USA, disproportionately favoured wealthy White residents, marginalising poorer Black communities (Article 88). International NGOs further intensified local exclusion through dominance in national and sub-national climate policy arenas (Article 105).

Thirdly, domination appeared through tokenistic participation strategies, falsely presenting inclusion without meaningful empowerment of marginalised groups (Articles 79, 91). This dynamic was clearly illustrated in gender affirmative quota policies, where women's formal involvement seldom translated into substantive decision-making power (Articles 97, 146). For example, the government of Nepal’s long-standing gender-quota policy for forest and water user committees failed to overcome societal gender norms that discouraged women’s active participation (Article 144). Indeed, entrenched patriarchal family structures and broader societal norms impeded women's meaningful engagement in decision-making processes across contexts such as India, Ghana, Kenya, and Namibia (Articles 67, 89, 146, 164). In India, systemic discrimination particularly affected widows and unmarried women, limiting their capacities to participate due to exclusionary inheritance practices and societal norms (Articles 36, 67, 146, 164).

The impacts of multi-layered domination were twofold. Numerous cases demonstrated how participatory processes reinforced rather than dismantled existing social hierarchies and power structures (Articles 47, 95, 128, 144). Participatory processes often failed to disrupt power dynamics around gender (Articles 76, 146), ethnicity (Article 69), race (Articles 123, 142), social class (Articles 123, 142), and caste (Articles 96, 116, 146, 162). Women were systematically excluded due to structural barriers and gender norms, limiting their effective involvement in climate adaptation and mitigation efforts (Articles 9, 13, 36, 47, 61, 89, 90, 146, 186).

Exclusion also persisted through poorly designed participatory processes, frequently inaccessible to disadvantaged groups (Articles 36, 47, 61, 80, 89, 90). International and national power structures, particularly in the Global South, routinely excluded local communities from meaningful engagement despite the stated participatory goals (Articles 105, 151, 172). This was exemplified by government-led relocation projects in Fiji, where communities experienced only the illusion of participatory decision-making and were ultimately coerced into relocation (Article 95).

Even initiatives explicitly designed as inclusive or co-productive could result in inequitable outcomes. Projects incorporating ILK through agricultural or drought-resilience practices often failed to achieve genuine power redistribution (Articles 77, 157). Local farmers frequently had minimal control over the dissemination and use of their knowledge, with academic institutions and NGOs dominating decisions on data sovereignty and management (Articles 25, 77, 133, 166). Such examples underline that the transformative potential of participatory co-production remains limited without explicit recognition and mitigation of underlying power asymmetries shaping knowledge processes.

Furthermore, elite stakeholders, including local political leaders, wealthy community members, traditional authorities, and representatives of international NGOs, exercised disproportionate control over participatory spaces, determining who participated, under what conditions, and with what outcomes. Elite capture significantly impeded the participation of disadvantaged groups, restricting their opportunities for input and decision-making, thus perpetuating historical patterns of inequity and marginalisation (Articles 90, 95, 121). For example, in a village-level agricultural initiative in rural Malawi (Article 9), village elders, scientists, agricultural experts, and government officials selected non-disabled men to participate in climate-resilient strategies, cementing social hierarchies through the exclusion of women, people with disabilities, and those living with HIV (Article 9). A plethora of studies highlights how women, primarily in the Global South, are routinely disadvantaged, silenced, and excluded within participatory processes, reflecting broader patterns of gender discrimination (Articles 9, 13, 36, 47, 61, 66, 67, 89, 90, 144, 186) operating within communities, cultures, and nations. Social discrimination in the form of ableism and health-related stigma (Articles 9, 67, 89, 90) similarly constrained participation opportunities for people with disabilities and individuals living with HIV or other medical conditions. Unsurprising given the evident barriers, there remains relatively limited research specifically examining how disability and (ill)health intersect with participation in climate mitigation and adaptation contexts. International NGOs and donors frequently contributed to these exclusionary dynamics, adopting paternalistic approaches that sidelined local priorities and imposed externally defined development agendas upon communities, particularly in Global South contexts (Articles 84, 90, 146). Meanwhile, issues of gender, ableism, and health stigma remain notably underexplored in studies from Global North contexts, with exceptions such as investigations into gender dynamics documented in Articles 50 and 120. The lack of attention to these forms of discrimination in the Global North literature does not imply their absence; rather, it highlights critical gaps within current research agendas.

### Participation in negotiation and contestation

Our review found that participation in efforts to strengthen mitigation and adaptation actions and foster climate resilience has often involved processes of negotiation and contestation, mainly to (re)address patterns of domination, marginalisation, and exclusion. However, negotiation processes were inevitably inscribed with power inequities. The reviewed literature indicated that participatory processes were generally created and maintained by powerful actors and institutions, which meant that local actor groups and/or disenfranchised peoples were rarely able to effectively negotiate with those in positions of authority (Articles 78, 80, 82, 83, 87, 95, 119, 160). Although negotiation processes had the potential to amplify voices and challenge injustices, this rarely occurred in practice due to persistent power asymmetries constraining these negotiation spaces (Articles 68, 86, 153). Moreover, communities experiencing multidimensional vulnerability often lacked the political capacities to influence decisions or meaningfully participate in negotiations (Articles 78, 160).

Participation through negotiation and contestation arises when actors explicitly seek to challenge power asymmetries, demand inclusion, or resist externally imposed climate policies. The extent to which these negotiations lead to substantive change, however, varies significantly between the Global North and Global South, and across mitigation and adaptation initiatives. In climate mitigation projects, contestation frequently emerges when governments or corporate actors implement top-down initiatives without meaningful local consultation or consideration of local rights. For instance, Indigenous and rural communities in Mexico and Brazil actively resisted large-scale wind and hydropower projects, citing dispossession, insufficient consultation, and environmental injustices (Articles 83, 119, 146). In these contexts, attempts at negotiation typically resulted in intensified conflict or repression, rather than peaceful resolution. Conversely, in the Global North, contestation in climate adaptation projects often unfolded through legal challenges or policy advocacy efforts, demonstrating incremental but meaningful shifts towards more inclusive governance. For example, urban flood resilience planning in Canada effectively integrated Indigenous knowledge following sustained advocacy from community organisations (Articles 69, 153).

Our findings demonstrate that participation does not necessarily progress linearly from exclusion to empowerment. Instead, participatory processes frequently involved iterative feedback loops, where initial efforts to increase involvement could reinforce existing power dynamics or gradually foster more inclusive governance models. In certain instances, initiatives starting as top-down or expert-led processes evolved through grassroots pressure into more contested spaces that facilitated broader community participation. Conversely, participation that appeared inclusive—such as stakeholder consultations—often did little to alter decision-making structures, thus reinforcing elite control through technocratic governance approaches. Recognising these iterative dynamics is crucial to designing participatory strategies that are genuinely adaptive rather than assuming linear progression towards empowerment.

Negotiation processes were frequently influenced or constrained by broader institutional power dynamics, further marginalising vulnerable and disadvantaged groups (Articles 49, 118). Negative outcomes included exacerbating existing community tensions or generating conflicts regarding community representation (Articles 10, 88, 90, 92, 132, 148). Deliberative forums often exposed intergenerational or intergroup disagreements regarding preferred adaptation strategies and future vulnerability reduction (Article 186). Moreover, concerns raised within formal participatory forums were often ignored, rendering these spaces vulnerable to elite capture (Articles 92, 99, 148).

### Participation in transformation and emancipation

Nevertheless, despite substantial evidence of participation having oppressive traits, some articles also demonstrate that participatory processes can be transformative and contribute to emancipatory outcomes (13%). Transformative participation requires fundamental shifts in governance, empowering marginalised communities to actively shape climate responses. Our findings indicate that transformative participation is documented more frequently in adaptation rather than mitigation projects, which tend to be dominated by state or corporate actors (Articles 69, 106, 133). Transformative participatory processes promote emancipation through power-sharing, supporting communities in recognising and addressing sources of inequality (Articles 9, 16, 29, 33, 41, 51, 53, 90, 112, 140). Such approaches include transferring decision-making responsibilities directly to communities (Article 41), creating collective cooperatives (Articles 49, 144), and openly addressing intersecting power inequalities (Article 112). Participatory processes prioritising vulnerable populations can significantly enhance community efficacy, rights awareness, and political confidence to engage actively in decision-making (Articles 58, 142).

Moreover, these transformative processes successfully highlighted and elevated community values, including common interests and shared goals (Articles 36, 94, 123, 142, 151). Several studies documented the emergence of innovative knowledge systems resulting from participatory engagements (Articles 3, 9, 30, 58, 77, 94, 99, 100, 103, 120, 121, 125, 154, 172). For example, Jackson et al.’s (2020, Article 36) project with Bedamuni communities in Papua New Guinea addressed power imbalances by reviving traditional governance and storytelling practices. Researchers collaborated with village leaders to encourage community members to use Indigenous narrative traditions within communal, gender-segregated spaces (longhouses) to reflect on local challenges and aspirations, aligning closely with socio-cultural norms (Article 36). This illustrates how decolonising methods foster meaningful participation by closely considering local contexts and historical practices.

Participation in decision-making processes often significantly contributed to transformative thinking and emancipation across diverse geographic and socio-cultural settings. Participation promoted lasting collaborations and co-production approaches (Articles 11, 25), encouraged inclusive and comfortable deliberative spaces (Article 30), improved social cohesion (Article 173), enhanced participant commitment (Article 35), and created spaces for collective innovation (Article 163). Participation proved essential for greater inclusion (Articles 9, 30, 130), empowerment (Articles 9, 49), and the mobilisation of alternative knowledge frameworks (Articles 58, 125). Nonetheless, several authors emphasised the necessity for researchers and practitioners to critically reflect on their positionalities and responsibilities in enabling transformative participation (Articles 14, 25, 29, 114).

Significantly, shifts towards political emancipation occurred when participatory processes deliberately prioritised marginalised individuals. Such efforts included explicit co-creation and deliberative spaces for vulnerable groups (Article 160), sensitivity to marginalised groups' adaptation needs (Article 162), and training and resource provision enabling effective participation in resilience-building initiatives (Articles 120, 162). Emancipatory participation was also strengthened by supporting knowledge- and rights-holders in challenging injustices (Article 106), fostering self-awareness of community capacities (Article 172), and critiquing and celebrating community values and achievements (Articles 35, 186). Moreover, inclusivity and reflexivity critically shaped participatory engagements, recognising pluralistic community values, aspirations, and multiple knowledge systems (Articles 25, 30, 36, 61, 94, 151), and acknowledging participants diverse objectives (Article 91). Emancipatory participation further demanded shared language and mutual understanding of key terminologies (Article 122), encouraging cross-sectoral and multi-scale interactions (Articles 116, 166, 173). Reflexive and iterative participatory learning processes—such as adaptive learning cycles, experiential pluralism, and facilitated dialogues—were crucial for transformational learning, including the unlearning of entrenched assumptions (Articles 29, 30, 66, 151). Creative experimentation and continuous learning were further identified as vital for novel governance approaches (Articles 133, 135).

Importantly, our analysis demonstrated the significance of who initiated and facilitated participation in shaping transformative outcomes. Studies frequently argued that inclusive participation initiated by historically marginalised social groups, rather than experts or elites, led to more substantial emancipatory impacts (Articles 58, 96, 103, 116, 133). For instance, in Bangladesh, as Sultana and Thompson (Article 113) reported, conflict among water users was resolved through a collaborative and cooperative community-based livelihood adaptation initiative that employed inclusive participatory methods, including facilitated dialogue, community meetings, and knowledge-sharing activities. The initiative actively engaged and empowered marginalised groups (elders and women) as equitable partners who possess unique local water knowledge and water management practices and who are well-positioned to contribute to the design and implementation of water management actions at a local level. The project outcomes directly benefitted women, unlike previous schemes that reinforced rather than challenged gender norms (Article 113).

Transformational change research highlights the iterative, reflexive nature of transformative learning (Articles 22, 106, 140). Transformative learning demands reconciling diverse community values and negotiating inevitable trade-offs and dilemmas (Article 30). Methods supporting transformational learning include community visioning processes, anticipatory and adaptive learning cycles, experiential pluralism, facilitated community dialogues, mutual aid practices, and collaborative scientific co-production with regular participatory consultations (Articles 9, 25, 30, 35, 36, 106, 140, 172).

Finally, effective emancipatory participation frequently involved reflexive knowledge-sharing across geographic scales (rural–urban, Global North–South), disciplinary boundaries, and various climate action spheres (mitigation, adaptation, and sustainable development) (Article 186). Systems of reflexivity were critical to illuminate participation barriers, highlighting both inclusive and exclusionary practices (Article 56). Numerous articles underscored participatory spaces enabling diverse knowledge exchanges and collaborative learning within and across scales (Articles 3, 6, 14, 22, 25, 33, 36, 58). Notable participatory methods for knowledge-sharing included formal committee meetings, community-driven roundtables, facilitated multi-stakeholder dialogues, experiential field experiments, and cross-border participatory networks (Articles 8, 69, 94, 100, 125, 154, 163, 166, 174). Furthermore, effective participatory approaches acknowledged and incorporated diverse ILK, especially within community-based adaptation, agroecology initiatives, and water management projects (Articles 50, 94, 154, 158, 162).

By analysing case studies from the Global North and Global South, our review advances a nuanced, pluralistic, and decolonial understanding of climate participation processes. Findings illustrate the limitations of linear, universalist participatory models that assume a straightforward progression from exclusion to empowerment. Instead, participation emerges as inherently contested and dynamic, shaped profoundly by historical legacies, institutional power structures, and prevailing socio-economic inequalities. Recognising this complexity highlights the urgent need for context-specific participatory approaches that transcend Western technocratic paradigms, embracing Indigenous governance frameworks, grassroots strategies of resistance, and diverse climate justice perspectives.

## Discussion

Our review critically analyses the climate mitigation and adaptation literature, focusing on participatory practices and their transformative potential. Our results reveal a spectrum of participatory practices, ranging from domination and exclusion to tokenistic, contestation and negotiation, and transformative approaches. We located participation processes along this continuum (Fig. 1) as it is a valuable way to see how participatory processes can reinforce or challenge power structures within climate adaptation and resilience efforts. However, as our results reveal, the extent to which participatory processes genuinely empower marginalised communities or perpetuate existing social inequities is highly variable.

### Participation as domination and exclusion

Our findings echo broader critiques that participatory practices often remain instrumental rather than transformative, frequently reinforcing existing power structures (McCarthy et al. 2018; Hügel and Davies 2020; Breyer and Mohr 2023). A significant issue emerging from our review is that participatory spaces typically limit the agency of marginalised actors. Governments, NGOs, and corporate actors routinely dominate participatory processes, reducing them to consultative exercises that legitimise predetermined decisions (Jiménez and Venegas 2023). This pattern is particularly pronounced in mitigation initiatives, such as REDD + and renewable energy projects, where local communities lack substantive influence (Avila-Calero 2017); similarly, climate adaptation initiatives often position communities merely as recipients of adaptation interventions rather than active decision-makers (Ober and Sakdapolrak 2020).

Although top-down, elite-driven participation occurs in both Global North and Global South contexts, exclusion operates distinctly across these regions. In Global South settings, participation is frequently state-controlled or donor-driven, with decision-making concentrated among government agencies, international NGOs, and private corporations. Conversely, Global North contexts typically frame participation as deliberative yet maintain highly technocratic processes dominated by experts and policymakers (Wamsler et al. 2020). In both cases, participation tends to legitimate rather than challenge existing power dynamics, constraining genuine public influence on decision-making.

Elite capture remains a persistent challenge (Sovacool et al. 2019; Araos 2023), as social elites—whether local leaders, corporate executives, or expert panels—exercise disproportionate influence within participatory spaces. However, elite involvement is not inherently problematic; many Indigenous and non-Western governance systems rely heavily on leadership from elders, chiefs, and knowledge holders whose exclusion can undermine the legitimacy and effectiveness of climate strategies (Chiblow 2020). For example, involving Māori elders and leaders in Aotearoa New Zealand ensures that mātauranga Māori (Māori knowledge), tikanga Māori (laws and protocols), and te ao Māori (worldviews) inform adaptation strategies, reflecting Indigenous governance structures (Smith 2020; Blackett et al. 2021). Hence, failing to recognise context-specific governance systems can lead to externally imposed participation rather than genuinely community-driven approaches (Bronen et al. 2020).

Another structural limitation identified is the embedding of participatory processes within neoliberal frameworks. These frameworks decentralise responsibilities to communities without adequate financial or political resources, resulting in a paradox of participation where communities are tasked with resilience-building but lack meaningful control over outcomes (Pelling et al. 2015; Sultana 2022). This critique aligns with broader discussions of climate coloniality, highlighting how Global North institutions shape adaptation and mitigation strategies in Global South contexts without adequately engaging local governance models, knowledge systems, or historical contexts (Ober and Sakdapolrak 2020).

The under-representation of marginalised groups, including Indigenous peoples, ethnic minorities, and women, reflects broader systemic inequalities within global institutions and academia (Ford et al. 2016; Wilkens and Datchoua-Tirvaudey 2022). Reducing vulnerability and fostering genuine resilience require recognising diverse knowledge systems, including those rooted in Indigenous ontologies and epistemologies (Whyte 2020b, 2020a). Hence, addressing climate coloniality and entrenched inequitable power dynamics through decolonising climate adaptation and resilience practices remains critically important for advancing equitable and genuinely participatory climate governance (Amo-Agyemang 2021; Sultana 2022).

### Negotiation and contestation in participatory processes

Without explicitly addressing power dynamics, participatory processes risk reproducing rather than challenging injustices (Baptiste and Devonish 2019). Scholars increasingly stress the need for participation to actively confront these dynamics and promote more equitable decision-making (Amorim-Maia et al. 2022; Garcia et al. 2022). Our findings highlight that the inclusiveness or exclusiveness of participation significantly depends on who sets the terms of engagement, whose voices are amplified, and whose knowledge is valued (Godden et al. 2021). Negotiation within participatory processes occurs when marginalised actors attempt to expand their influence in decision-making spaces; yet, these efforts are often constrained by existing governance structures and elite control (Pelling et al. 2015; Nightingale 2017).

When negotiation fails to secure meaningful engagement, contestation frequently arises as communities respond to perceived unjust policies and exclusionary governance structures (Wilkens and Datchoua-Tirvaudey 2022). Contestation takes diverse forms, including policy advocacy, legal challenges, and direct resistance or civil disobedience, shaped significantly by socio-cultural and political contexts (Cervera-Marzal et al. 2019; Cha 2020). Although contestation holds potential to disrupt exclusionary practices, it is frequently met with institutional inertia, co-optation, or outright repression, particularly when marginalised groups challenge dominant socio-economic and governance systems (Avila-Calero 2017).

Resistance to imposed climate policies does not always produce greater inclusivity; rather, it often reinforces power hierarchies by framing affected communities as obstacles to progress instead of legitimate stakeholders (Whyte 2020b). Grassroots movements, particularly those challenging closely aligned state and corporate interests, often face criminalisation and delegitimisation, reflecting differential responses between Global North and South contexts (Scheidel et al. 2020; Temper et al. 2020; Gonda et al. 2023). Community-led resistance in the Global North is frequently interpreted as civic engagement, whereas similar actions in the Global South commonly trigger surveillance or state-backed repression (Gonda et al. 2023). Despite these challenges, contestation remains crucial for asserting community perspectives, building solidarity, and pressuring institutions towards more inclusive governance practices (LeQuesne 2019; James and Mack 2020; Whyte 2020a).

Dominant narratives within resilience scholarship typically frame conflict negatively, emphasising its destructive effects on communities and infrastructure (Vivekananda et al. 2014). However, participatory processes that prioritise conflict avoidance risk reinforcing inequities rather than addressing them (Tschakert et al. 2023). Indeed, dissent and conflict within participatory spaces can reveal underlying injustices, fostering social connections, collective identity, and solidarity that strengthen resilience (Harris et al. 2018). Recent scholarship calls for explicit acknowledgement of power asymmetries, arguing that contestation is integral rather than indicative of failure within participatory approaches (Kesby 2005; Gonda et al. 2023).

Instead of smoothing over conflicts in pursuit of consensus, participatory frameworks should actively accommodate and respond to contestation as legitimate civic engagement. Researchers and decision-makers must acknowledge participation as inherently political, shaped by struggles over power, representation, and knowledge (Mason and Niewolny 2022). Negotiation and contestation should not be viewed as mutually exclusive but as dynamic points along a continuum, with contested spaces potentially evolving into more negotiated or transformative forums (Le Heron et al. 2019; Garcia and Tschakert 2022). Reframing participation as an evolving political process rather than a neutral mechanism can thus challenge structural inequalities and meaningfully advance climate justice (Wojciechowska 2022).

### Transformative and emancipatory participation

The transformative potential of participatory processes lies in their ability to challenge power structures and foster inclusive decision-making. While participatory approaches are often framed as mechanisms for achieving equity and justice in climate mitigation and adaptation, our findings (paralleling wider scholarship) suggest that participation does not inherently lead to empowerment or transformation (Turnhout et al. 2020). Rather, transformative participation necessitates an explicit commitment to redistributing power, amplifying marginalised voices, including diverse knowledge systems, and fostering structural changes (Zurba et al. 2019). It must go beyond surface-level inclusion to actively disrupt systems of exclusion and dispossession that underpin social and climate injustices (Osborne 2015).

While capable of fostering collective action, participatory processes often remain constrained by neoliberal governance structures (Robinson and van Veelen 2022). This dynamic risks reinforcing climate responsibilisation, the practice whereby individuals, households, or local communities bear the burden of mitigation and adaptation actions while state and corporate actors retain control over climate finance and policies (Döbbe and Cederberg 2024). A truly transformative approach to participation would require actions that challenge these structural inequities and ensure that marginalised groups have access to resources, decision-making authority, and the ability to set their adaptation and mitigation priorities based on their values, ways of life, aspirations for future generations, and visions of climate justice (Amorim-Maia et al. 2022).

Finally, scholarship on participation and emancipation highlights that meaningful transformation is more likely to occur when participatory processes are rooted in alternative political imaginaries that challenge the status quo (Mason and Niewolny 2022). Emancipation in the context of participation means going beyond mere inclusion, consultation, or negotiation and involves actions aimed at fundamentally challenging and transforming the socio-political, economic, and political structures that perpetuate inequities and injustices. Indeed, Shi and Moser (2021) identify that “empowered and deliberative” participatory practices are critically needed to assert the normative values of inclusion, justice, and equity and foster large-scale transformative systemic thinking that feeds into just mitigation and adaptation policies, practices, and resource flows. Interweaving participation with emancipation moves mitigation and adaptation beyond mere technical fixes (Nightingale et al. 2019) and framing it as a transformative process that reconfigures how power is distributed in climate governance and on-the-ground actions (Garcia and Tschakert 2022).

Public engagement with climate imaginaries that seek to go beyond adaptation, mitigation, and resilience beyond technocratic, carbon markets, and consumer-centred approaches is already taking place (Shi and Moser 2021). Participatory processes can further shift the focus towards collective care, solidarity, and justice strategies and engendering just mitigation and adaptation policies and actions. Participatory frameworks incorporating multi-species justice, relational ontologies, and Indigenous environmental ethics create new possibilities for genuinely transformative climate action (Tschakert et al. 2021). However, meaningful transformation depends on directly confronting entrenched colonial, patriarchal, and capitalist structures that continue to shape climate mitigation, adaptation, and governance rather than positioning participation as a neutral or apolitical tool (Groves et al. 2021).

A key distinction must be made between empowerment and emancipation in participatory processes. While empowerment involves increasing agency within existing systems (Auger et al. 2016), emancipation demands reconfiguring the systems themselves to address structural inequities (Manuel-Navarrete 2010). To this end, emancipatory approaches to climate mitigation, adaptation, and resilience aim to transform the systems (social, cultural, and political) to reduce vulnerability, enhance the capacity to mitigate emissions (Einecker and Kirby 2020), build resilience among the most disadvantaged groups, and foster transformative adaptation (Garcia and Tschakert 2022). Shi and Moser (2021) identify that “empowered and deliberative” participatory practices are critically needed to assert the normative values of inclusion, justice, and equity and foster large-scale transformative systemic thinking that feeds into just mitigation and adaptation policies, practices, and resource flows. Accordingly, interweaving participation with empowerment and emancipation is intended to move mitigation and adaptation beyond technical fixes (Nightingale et al. 2019). Transformative participation thus requires shifting from consultation and negotiation to co-creation, co-design, and co-governance approaches, ensuring that marginalised groups are not merely engaged in decision-making but have meaningful authority over climate actions and resource allocations (Garcia and Tschakert 2022). This aligns with broader calls to decolonise participation and governance (Wilkens and Datchoua-Tirvaudey 2022), which includes recognition of Indigenous sovereignty (Eitzel et al. 2020).

Researchers have stressed that transformative participation must be iterative, reflexive, and contextual (Tschakert et al. 2023); this means moving beyond one-off engagements (such as a public consultation phase) towards sustained processes of participation that involves learning, adjusting, and collective action. Research on co-production, for instance, highlights that effective participatory processes require spaces of mutual learning where different knowledge systems are valued and actively shape decision-making (Bronen et al. 2020). However, the challenge remains that many participatory initiatives, particularly those funded by external agencies, retain extractive logic, where communities contribute knowledge but lack decision-making power over how that knowledge is used or whose priorities take precedence (Malloy and Ashcraft 2020).

Thus, while the transformative potential of participation is well-documented (Mehta et al. 2019), its realisation depends on who holds power, whose knowledge is prioritised, and whether participatory processes actively destabilise or reinforce structural inequities (Shi and Moser 2021). Moving towards emancipatory participation requires a radical rethinking of governance structures, knowledge systems, funding arrangements, and decision-making processes, ensuring that participation is not simply a mechanism for inclusion (in an instrumental manner) but a means of redistributing power and enacting climate justice (Amorim-Maia et al. 2022).

Despite growing attention to multi-species justice (Tschakert et al. 2021), our review identified no adaptation, mitigation, or resilience studies addressing more-than-human participation (Sheikh et al. 2023). The absence of more-than-human considerations likely reflects the enduring anthropocentrism within the fields (Tschakert et al. 2021), where decision-making frameworks prioritise human interests, often overlooking the entanglements between humans and more-than-humans in shaping resilience (Yates et al. 2023). Expanding participatory approaches to acknowledge and include the agency of plants, animals, and other more-than-human entities could provide opportunities to foster deeper, relational understandings of climate mitigation, adaptation, and resilience. Indeed, such approaches would benefit Indigenous-led and community-led initiatives (Rarai et al. 2022). Future research should critically examine how participation might extend beyond humans to recognise and consider the rights, needs, and contributions of more-than-human beings within climate mitigation, adaptation, and resilience (Bastian et al. 2016).

## Conclusion

Our review outlines a continuum of participation, from domination and exclusion to transformation and emancipation, highlighting the diverse ways participatory processes shape climate mitigation, adaptation, and resilience-building. While participation is commonly promoted as essential for equitable climate governance, our findings demonstrate it often reinforces existing power structures through tokenistic methods, elite control, and exclusionary practices. Nevertheless, examples of negotiation, contestation, and transformative participation offer valuable insights for designing more effective and just participatory processes.

Realising genuine transformation requires moving beyond consultation towards significant shifts in power, investing in ongoing practices such as co-design, co-production, and community-led decision-making. This necessitates directly challenging the colonial, capitalist, and patriarchal structures that shape climate governance rather than viewing participation as politically neutral. Moreover, participatory frameworks must expand to integrate intergenerational justice, Indigenous sovereignty, and relational governance models that value diverse knowledge systems. Our review identified an absence of studies addressing multi-species justice or more-than-human participation despite increasing acknowledgement of their relevance for transformative climate action.

Future research should explore transformative and emancipatory participatory approaches that are inclusive, reflexive, politically engaged, and capable of addressing the underlying causes of vulnerability, marginalisation, and climate injustice(s). This may include examining how to meaningfully incorporate diverse voices and knowledge systems, leveraging conflict as a catalyst for positive social change, and examining governance models prioritising climate justice and multi-species justice. Empirical research is primarily needed to investigate how participatory processes can be structured to enhance political agency, build long-term resilience, and contribute to climate justice in ways that are not only inclusive but also emancipatory.

## Supplementary Information

Below is the link to the electronic supplementary material.

Supplementary file1 (PDF 2478 KB)

## Data Availability

The data supporting the findings of this paper are not publicly available due to the nature of the systematic review, which relies exclusively on analysis and synthesis of published materials already accessible through existing scholarly databases. Additionally, no new empirical or primary datasets were generated or analysed in this research. Therefore, sharing a separate database would be redundant, as all sources reviewed can be independently accessed through standard academic literature searches.
