# Supplementary material for: Participatory approaches to climate adaptation, resilience, and mitigation: A systematic review
Source: Ambio. 2025 Jun 4;54(12):2005–20. doi: 10.1007/s13280-025-02202-z (PMC12569303; doi:10.1007/s13280-025-02202-z)
Supplement: Supplementary file 1 — Supplementary file1 (PDF 2478 KB) [file 13280_2025_2202_MOESM1_ESM.pdf]

Supplementary Material for the Article: "Participatory approaches to climate adaptation, resilience, and mitigation: a systematic review"

Journal: Ambio

Authors: Meg Parsons*, School of Environment, The University of Auckland, 1142 Auckland New Zealand, https://orcid.org/0000-0001-8721-659X

* Corresponding author: Email: meg.parsons@auckland.ac.nz

Naomi Joy Godden

Naomi Joy Godden, Centre for People, Place and Planet and School of Arts and Humanities, Edith Cowan University, https://orcid.org/0000-0001-9881-3365

Karen Paiva Henrique ('last name: Henrique', 'first name: Karen Paiva' for publications), Department of Human Geography, Planning and International Development, University of Amsterdam, https://orcid.org/0000-0002-2163-2854

Petra Tschakert, Faculty of Humanities, School of Media, Creative Arts and Social Inquiry, Curtin University, Bentley, Western Australia https://orcid.org/0000-0002-4268-3378

Noémi Gonda Department of Urban and Rural Development, Swedish University of Agricultural Sciences,

https://orcid.org/0000-0002-1261-8380

Ed Atkins, School of Geographical Sciences, University of Bristol, http://orcid.org/0000-0001-8694-8465

Karin Steen, Lund University Centre for Sustainability Studies, Lund University, https://orcid.org/0000-0003-4176-4296

Roa Petra Crease (first name: Roa Petra, last name: Crease), School of Environment, The University of Auckland, https://orcid.org/0000-0001-7790-7872

Supplementary Material

Online Resource: Detailed Methodology

We conducted a systematic review of literature (2015–2020), utilizing keywords related to participation, climate change, and power. The detailed protocol and methods are provided in Supplementary Information (see Figure S1). Our six-stage process (see Figure S1) began by framing our guiding research question: *How do participatory practices in climate mitigation and/or adaptation reinforce or challenge power structures, and what transformative potential do they hold?* Next, we established inclusion criteria focusing on peer-reviewed articles from 2015–2020. The time frame (2015–2020) was chosen to capture the most recent theoretical and empirical contributions in the post-Paris Agreement era, where participatory decision-making processes have been increasingly promoted as a pillar of climate mitigation and adaptation policies. Stage 3 involved creating a search protocol that we applied to a single academic database (Scopus) and conducting our initial search. The author team selected Scopus due to its interdisciplinary coverage, which includes an extensive range of peer-reviewed publications covering humanities, social sciences, natural sciences, and transdisciplinary studies), and its accessibility across our international team (all team members could access it) (see Figure S1 and Figure S2). Despite its strengths, a key limitation of the systematic review method is its reliance on Scopus-indexed, English-language publications, which may exclude Indigenous and non-Western knowledge systems often published in alternative formats or languages (Whyte 2020b). This limitation highlights the need for future participatory research that actively engages with other databases and non-academic sources.

Figure S1: Six-staged process (Diagram showing the six stages of the data collection and analysis process, which is described in text).

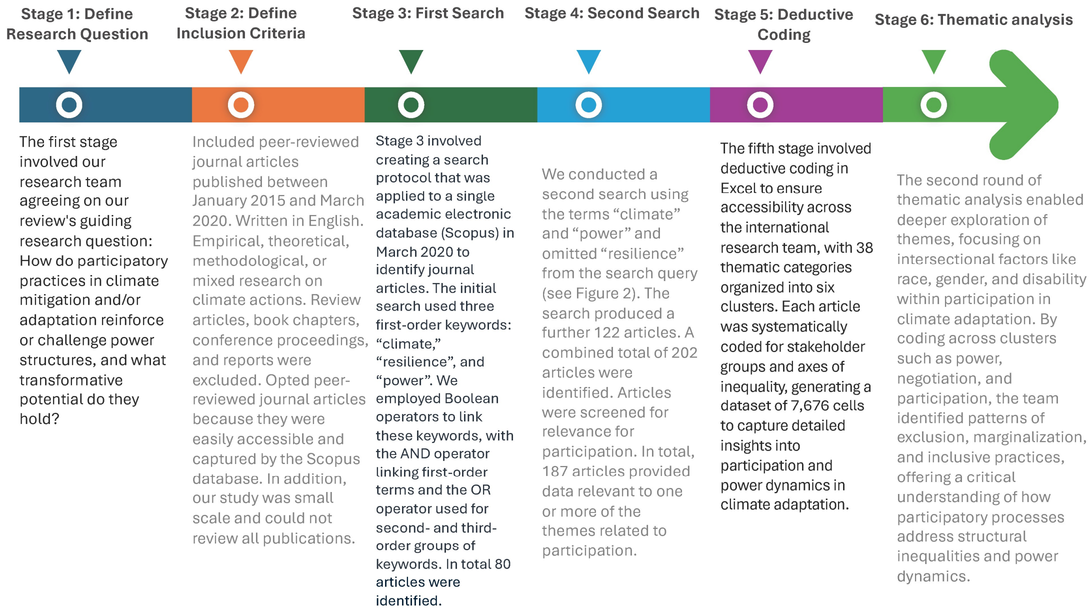

Figure S2: Diagram last three stages of the data collection process

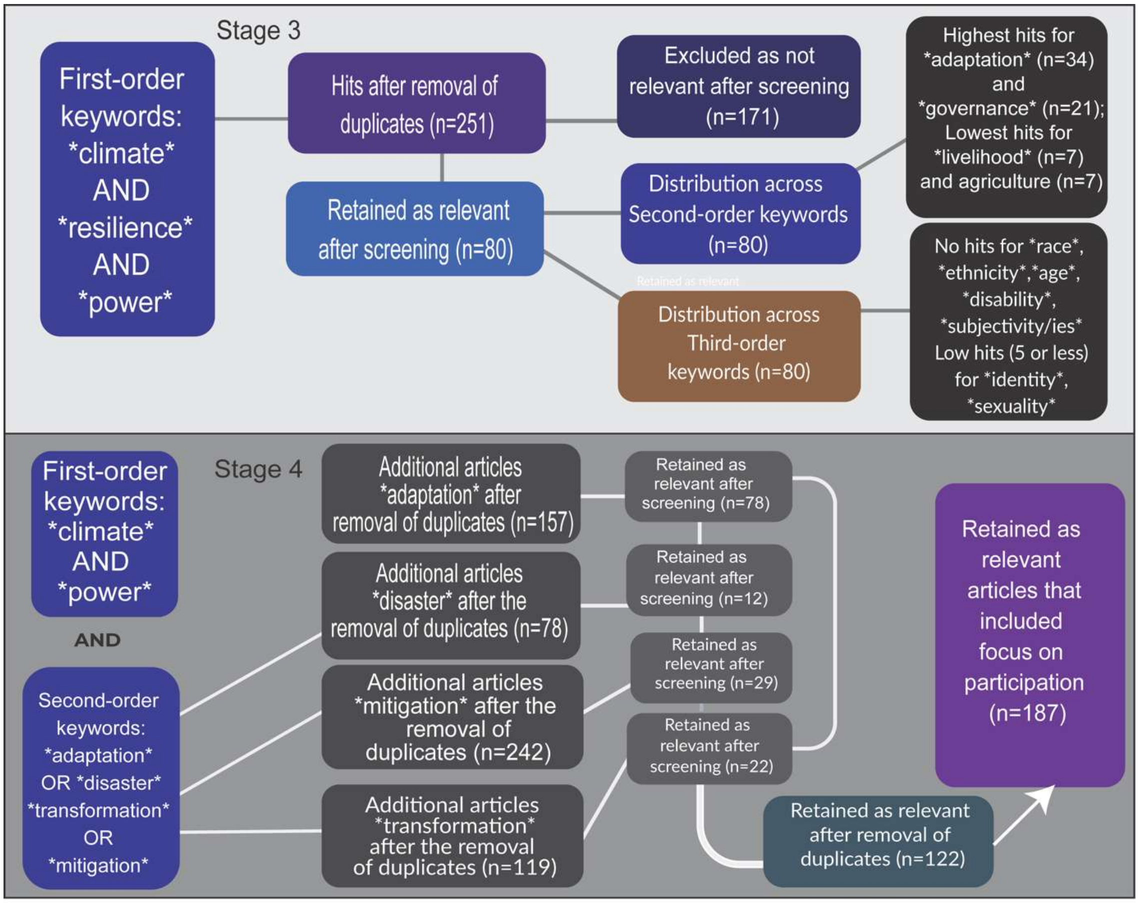

We began our search (Stage 3, see Figure S2) using three groups of keywords that reflect our guiding question (Table S1). Keywords were combined using Boolean operators to maximise search sensitivity while maintaining relevance. The first-order keywords ("climate" AND "power" AND "resilience") were combined with second-order terms (adaptation, mitigation, transformation, vulnerability, disaster, governance, etc.) using "OR" to capture a broad scope. The third-order keywords (race, gender, class, knowledge, rights, participation) were added using "AND" to refine the focus on power dynamics and participatory processes. We used a wide range of keywords to identify relevant articles; however, our review prioritised articles that focused on participatory processes within climate actions, including adaptation and mitigation efforts. The initial search produced a total of 80 articles.

Table S1. Keywords used to search through Scopus

**First-order keywords that are central concepts for this review**	*climate* AND *power* AND *resilience*
**Second-order keywords that provide coverage of the main themes**	*adaptation* OR *mitigation* OR *transformation* OR *disaster* OR *vulnerability* OR *capacity* OR *governance* OR *security* (water, food) OR *livelihood(s)* OR *rural* OR *urban* OR *agricultural*
**Third-order keywords for power and inequalities dimensions**	*race* OR *ethnicity* OR *gender* OR *class*, OR *age* OR *indigenous*, OR *disability*, OR *justice* OR *participation*, OR *identity* OR *inequality* OR *stakeholder* OR *conflict* OR *rights* OR *knowledge* OR *authority OR *subjectivity/ies*

To address gaps in the initial search, we conducted a second search (Stage 4) of Scopus using only “climate” and “power” as keywords, omitting “resilience” from the search query. The omission of "resilience" from the search query resulted in 122 new articles. It should be noted that "resilience" is frequently used in ecological and engineering studies that are purely technical and do not involve participatory approaches. In contrast, climate change adaptation and mitigation sometimes do not use the term resilience and instead prefer terms like adaptive capacity and capabilities. The discrepancy in terminology reflects disciplinary differences in how different terms are operationalised and necessitated us conducting another search. The second search helped to identify studies that focused on the underrepresented dimensions of power (e.g., race, gender, disability), as shown in Figure S1.

Our initial search employed a detailed keyword strategy combining three keyword groups through Boolean operators (see Table S1). Keywords included "climate," "power," and "resilience," complemented by secondary terms like "adaptation," "mitigation," and "governance," and refined using intersectional factors such as "race," "gender," and "participation." This initial query yielded 80 relevant articles. Recognising disciplinary differences in terminology, particularly the term "resilience", often appearing in technical studies without participatory elements, we conducted a second search excluding "resilience," resulting in 122 additional articles, totalling 202. After reading through the papers, we narrowed our dataset to 187 papers, which explicitly addressing participatory processes. Thus our final dataset consisted of 187 papers listed in Table S2.

Table S2:

Number assigned to article	Full Reference
1	Ramalho, J. 2019. Empowerment in the era of resilience-building: gendered participation in community-based (disaster) risk management in the Philippines. *International Development Planning Review* 41. Liverpool University Press (UK): 129–149. doi:10.3828/idpr.2018.25.
2	Hirons, M., Z. Mehrabi, T. A. Gonfa, A. Morel, T. W. Gole, C. McDermott, E. Boyd, E. Robinson, et al. 2018. Pursuing climate resilient coffee in Ethiopia – A critical review. *Geoforum* 91: 108–116. doi:10.1016/j.geoforum.2018.02.032.
3	Fisher, S., and D. Dodman. 2019. Urban climate change adaptation as social learning: Exploring the process and politics. *Environmental Policy and Governance* 29: 235–247. doi:10.1002/eet.1851.
4	Kaika, M. 2017. 'Don't call me resilient again!': the New Urban Agenda as immunology … or … what happens when communities refuse to be vaccinated with 'smart cities' and indicators. *Environment and Urbanization* 29. SAGE Publications Ltd: 89–102. doi:10.1177/0956247816684763.
5	Mikulewicz, M. 2019. Thwarting adaptation's potential? A critique of resilience and climate-resilient development. *Geoforum* 104: 267–282. doi:10.1016/j.geoforum.2019.05.010.
6	Dixon, J. L., and L. C. Stringer. 2015. Towards a Theoretical Grounding of Climate Resilience Assessments for Smallholder Farming Systems in Sub-Saharan Africa. *Resources* 4. Multidisciplinary Digital Publishing Institute: 128–154. doi:10.3390/resources4010128.
7	Alvarez, M. K., and K. Cardenas. 2019. Evicting Slums, 'Building Back Better': Resiliency Revanchism and Disaster Risk Management in Manila. *International Journal of Urban and Regional Research* 43: 227–249. doi:10.1111/1468-2427.12757.
8	Terävainen, T. 2019. Negotiating Water and Technology—Competing Expectations and Confronting Knowledges in the Case of the Coca Codo Sinclair in Ecuador. *Water* 11: 411. doi:10.3390/w11030411.

9	Kerr, R. B., H. Nyantakyi-Frimpong, L. Dakishoni, E. Lupafya, L. Shumba, I. Luginaah, and S. S. Snapp. 2018. Knowledge politics in participatory climate change adaptation research on agroecology in Malawi. *Renewable Agriculture and Food Systems* 33. Cambridge University Press: 238–251. doi:10.1017/S1742170518000017.
10	McDonnell, S. 2019. Other Dark Sides of Resilience: Politics and Power in Community-Based Efforts to Strengthen Resilience. *Anthropological Forum* 0. Routledge: 1–18. doi:10.1080/00664677.2019.1647828.
11	Conca, K. 2019. Prospects for a multi-stakeholder dialogue on climate engineering. *Environmental Politics* 28. Routledge: 417–440. doi:10.1080/09644016.2018.1522065.
12	Bizikova, L., S. Tyler, M. Moench, M. Keller, and D. Echeverria. 2016. Climate resilience and food security in Central America: a practical framework. *Climate and Development* 8: 397–412. doi:10.1080/17565529.2015.1064806.
13	Eriksen, S. H., L. K. Cramer, I. Vetrhus, and P. Thornton. 2019. Can Climate Interventions Open Up Space for Transformation? Examining the Case of Climate-Smart Agriculture (CSA) in Uganda. *Frontiers in Sustainable Food Systems* 3. Frontiers. doi:10.3389/fsufs.2019.00111.
14	Wijsman, K., and M. Feagan. 2019. Rethinking knowledge systems for urban resilience: Feminist and decolonial contributions to just transformations. *Environmental Science & Policy* 98: 70–76. doi:10.1016/j.envsci.2019.04.017.
15	Taylor, M., and S. Bhasme. 2020. Between deficit rains and surplus populations: The political ecology of a climate-resilient village in South India. *Geoforum*. doi:10.1016/j.geoforum.2020.01.007.
16	Rotz, S., and E. D. G. Fraser. 2015. Resilience and the industrial food system: analyzing the impacts of agricultural industrialization on food system vulnerability. *Journal of Environmental Studies and Sciences* 5: 459–473. doi:10.1007/s13412-015-0277-1.
17	Mirumachi, N., A. Sawas, and M. Workman. 2020. Unveiling the security concerns of low carbon development: climate security analysis of the undesirable and unintended effects of mitigation and adaptation. *Climate and Development* 12. Taylor & Francis: 97–109. doi:10.1080/17565529.2019.1604310.
18	Weinstein, L., A. Rumbach, and S. Sinha. 2019. Resilient Growth: Fantasy Plans and Unplanned Developments in India’s Flood-Prone Coastal Cities. *International Journal of Urban and Regional Research* 43: 273–291. doi:10.1111/1468-2427.12743.

18	Tanyag, M. 2018. Resilience, Female Altruism, and Bodily Autonomy: Disaster-Induced Displacement in Post-Haiyan Philippines. *Signs: Journal of Women in Culture and Society* 43: 563–585. doi:10.1086/695318.
19	Sherren, K., L. Loik, and J. A. Debner. 2016. Climate adaptation in 'new world' cultural landscapes: The case of Bay of Fundy agricultural dykelands (Nova Scotia, Canada). *Land Use Policy* 51: 267–280. doi:10.1016/j.landusepol.2015.11.018.
20	Derickson, K. D. 2018. Urban geography III: Anthropocene urbanism. *Progress in Human Geography* 42. SAGE Publications Ltd: 425–435. doi:10.1177/0309132516686012.
21	Reinecke, S., and M. Blum. 2018. Discourses across Scales on Forest Landscape Restoration. *Sustainability (Switzerland)* 10. Scopus. doi:10.3390/su10030613.
22	Colenbrander, S., D. Dodman, and D. Mitlin. 2018. Using climate finance to advance climate justice: the politics and practice of channelling resources to the local level. *Climate policy* 18. Taylor & Francis: 902–915. doi:10.1080/14693062.2017.1388212.
23	Christoplos, I., and C. McGinn. 2016. Climate Change Adaptation from a Human Rights Perspective: Civil Society Experiences in Cambodia. *Forum for Development Studies* 43. Routledge: 437–461. doi:10.1080/08039410.2016.1199443.
24	Castán Broto, V., and L. K. Westman. 2020. Ten years after Copenhagen: Reimagining climate change governance in urban areas. *WIREs Climate Change* 11: e643. doi:10.1002/wcc.643.
25	Grabowski, Z. J., P. Z. Klos, and C. Monfreda. 2019. Enhancing urban resilience knowledge systems through experiential pluralism. *Environmental Science & Policy* 96: 70–76. doi:10.1016/j.envsci.2019.03.007.
26	Anderson, C. R., J. Bruil, M. J. Chappell, C. Kiss, and M. P. Pimbert. 2019. From Transition to Domains of Transformation: Getting to Sustainable and Just Food Systems through Agroecology. *Sustainability* 11. Multidisciplinary Digital Publishing Institute: 5272. doi:10.3390/su11195272.
27	Carmody, P., and D. Taylor. 2016. Globalization, Land Grabbing, and the Present-Day Colonial State in Uganda: Ecolonization and Its Impacts. *The Journal of Environment & Development* 25. SAGE Publications Inc: 100–126. doi:10.1177/1070496515622017.

28	Rumbach, A. 2016. Decentralization and small cities: Towards more effective urban disaster governance? *Habitat International* 52. Decentralising Disaster Governance in Urbanising Asia: 35–42. doi:10.1016/j.habitatint.2015.08.026.
29	Westling, E. L., L. Sharp, D. Scott, S. Tait, M. Rychlewski, and R. M. Ashley. 2019. Reflexive adaptation for resilient water services: Lessons for theory and practice. 30*Global Environmental Change* 57. Scopus. doi:10.1016/j.gloenvcha.2019.101937.
30	Tschakert, P., N. Tuana, H. Westskog, B. Koelle, and A. Afrika. 2016. T CHANGE : the role of values and visioning in transformation science. *Current Opinion in Environmental Sustainability* 20: 21–25. doi:10.1016/j.cosust.2016.04.003.
31	Ajibade, I. 2017. Can a future city enhance urban resilience and sustainability? A political ecology analysis of Eko Atlantic city, Nigeria. *International Journal of Disaster Risk Reduction* 26. Africa's Urban Risk and Resilience: 85–92. doi:10.1016/j.ijdrr.2017.09.029.
32	Kmoch, L., T. Pagella, M. Palm, and F. Sinclair. 2018. Using local agroecological knowledge in climate change adaptation: A study of tree-based options in Northern Morocco. *Sustainability (Switzerland)* 10. Scopus. doi:10.3390/su10103719.
33	Cho, M.-R. 2020. Urban resilience through progressive governance: The case of the 'One Less Nuclear Power Plant' policy, Seoul, Korea. *Urban Studies* 57. SAGE Publications Ltd: 1434–1451. doi:10.1177/0042098019838965.
34	Fainstein, S. 2015. Resilience and Justice. *International Journal of Urban and Regional Research* 39: 157–167. doi:10.1111/1468-2427.12186.
35	Fernandes-Jesus, M., A. Carvalho, L. Fernandes, and S. Bento. 2017. Community engagement in the Transition movement: views and practices in Portuguese initiatives. *Local Environment* 22. Routledge: 1546–1562. doi:10.1080/13549839.2017.1379477.
36	Jackson, G., K. E. McNamara, and B. Witt. 2020. "System of hunger": Understanding causal disaster vulnerability of indigenous food systems. *Journal of Rural Studies* 73: 163–175. doi:10.1016/j.jrurstud.2019.10.042.
37	Harvey, B., L. Jones, L. Cochrane, and R. Singh. 2019. The evolving landscape of climate services in sub-Saharan Africa: What roles have NGOs played? *Climatic Change* 157: 81–98. doi:10.1007/s10584-019-02410-z.
38	Potter, K., and T. Vilcan. 2020. Managing urban flood resilience through the English planning system: insights from the 'SuDS-face.' *Philosophical Transactions of the Royal Society A: Mathematical, Physical and Engineering Sciences* 378. Royal Society: 20190206. doi:10.1098/rsta.2019.0206.

39	Betteridge, B., and S. Webber. 2019. Everyday resilience, reworking, and resistance in North Jakarta's kampungs. *Environment and Planning E: Nature and Space* 2. SAGE Publications Ltd STM: 944–966. doi:10.1177/2514848619853985.
40	Archer, D. 2015. Making capacity building critical: Power and justice in building urban climate resilience in Indonesia and Thailand. *Urban Climate* 14. International Institute for Environment and Development, London: 68–78. doi:10.1016/j.uclim.2015.06.007.
41	Sapkota, P., R. J. Keenan, and H. R. Ojha. 2019. Co-evolving dynamics in the social-ecological system of community forestry––prospects for ecosystem-based adaptation in the Middle Hills of Nepal. *Regional Environmental Change* 19: 179–192. doi:10.1007/s10113-018-1392-9.
42	Herrera, H. 2017. Resilience for Whom? The Problem Structuring Process of the Resilience Analysis. *Sustainability* 9. Multidisciplinary Digital Publishing Institute: 1196. doi:10.3390/su9071196.
43	McNeeley, S. M. 2017. Sustainable Climate Change Adaptation in Indian Country. *Weather Climate and Society* 9: 392–403. doi:10.1175/WCAS-D-16-0121.1.
44	Petzold, J., B. M. W. Ratter, and A. Holdschlag. 2018. Competing knowledge systems and adaptability to sea-level rise in The Bahamas. *Area* 50. John Wiley & Sons, Ltd: 91–100. doi:10.1111/area.12355.
45	Steenberg, J. W. N., M. A. Timm, K. L. Laurent, K. B. Friedman, G. Krantzberg, D. Scavia, and I. F. Creed. 2015. Living on the Edge: How we converted challenges into profitable opportunities. *Journal of Great Lakes Research* 41. The Great Lakes Futures Project: Using Scenario Analysis to Develop a Sustainable Socio-Ecologic Vision for the Great Lakes-St. Lawrence River Basin: 150–160. doi:10.1016/j.jglr.2014.12.001.
46	Hannaford, M. J. 2018. Long-term drivers of vulnerability and resilience to drought in the Zambezi-Save area of southern Africa, 1505–1830. *Global and Planetary Change* 166: 94–106. doi:10.1016/j.gloplacha.2018.05.001.
47	Nagoda, S., and A. J. Nightingale. 2017. Participation and Power in Climate Change Adaptation Policies: Vulnerability in Food Security Programs in Nepal. *World Development* 100: 85–93. doi:10.1016/j.worlddev.2017.07.022.
48	Nisbet, M. C. 2018. Strategic philanthropy in the post-Cap-and-Trade years: Reviewing U.S. climate and energy foundation funding. *WIREs Climate Change* 9: e524. doi:10.1002/wcc.524.

49	Tschakert, P., P. J. Das, N. Shrestha Pradhan, M. Machado, A. Lamadrid, M. Buragohain, and M. A. Hazarika. 2016. Micropolitics in collective learning spaces for adaptive decision making. *Global Environmental Change* 40: 182–194. doi:10.1016/j.gloenvcha.2016.07.004.
50	Fazey, I., E. Carmen, F. S. Chapin III, H. Ross, J. Rao-Williams, C. Lyon, I. L. C. Connon, B. A. Searle, et al. 2018. Community resilience for a 1.5 °C world. *Current Opinion in Environmental Sustainability* 31: 30–40. Scopus. doi:10.1016/j.cosust.2017.12.006.
51	Ensor, J., J. Forrester, and N. Matin. 2018. Bringing rights into resilience: revealing complexities of climate risks and social conflict. *Disasters* 42: S287–S305. doi:10.1111/disa.12304.
52	Córdoba Vargas, C. A., S. Hortúa Romero, and T. León Sicard. 2020. Key points of resilience to climate change: a necessary debate from agroecological systems. *Climate and Development* 12. Taylor & Francis: 564–574. doi:10.1080/17565529.2019.1664376.
53	Andersson, E., E. C. H. Keskitalo, and S. Bergstén. 2018. In the eye of the storm: adaptation logics of forest owners in management and planning in Swedish areas. *Scandinavian Journal of Forest Research* 33. Taylor & Francis: 800–808. doi:10.1080/02827581.2018.1494305.
54	De Roeck, F. 2019. Governmentality and the climate-development nexus: The case of the EU Global Climate Change Alliance. *Global Environmental Change* 55: 160–167. doi:10.1016/j.gloenvcha.2019.02.006.
55	de Abreu-Mota, M. A., R. P. Medeiros, and M. A. Noernberg. 2018. Resilience thinking applied to fisheries management: perspectives for the mullet fishery in Southern-Southeastern Brazil. *Regional Environmental Change* 18: 2047–2058. doi:10.1007/s10113-018-1323-9.
56	Lawless, C. J. 2018. Exploring the socio-material boundaries of climate change resilience. *Environmental Sociology* 4. Routledge: 434–444. doi:10.1080/23251042.2018.1449341.
57	Blackburn, S. 2018. What Does Transformation Look Like? Post-Disaster Politics and the Case for Progressive Rehabilitation. *Sustainability* 10. Multidisciplinary Digital Publishing Institute: 2317. doi:10.3390/su10072317.
58	Otero, I., M. Castellnou, I. González, E. Arilla, L. Castell, J. Castellví, F. Sánchez, and J. Ø. Nielsen. 2018. Democratizing wildfire strategies. Do you realize what it means? Insights from a participatory process in the Montseny region (Catalonia, Spain). *PLOS ONE* 13. Public Library of Science: e0204806. doi:10.1371/journal.pone.0204806.

59	Leap, B. 2018. Not a zero-sum game: inequalities and resilience in Sumner, Missouri, the Gooseless Goose Capital of the World. *Gender, Place & Culture* 25. Routledge: 288–308. doi:10.1080/0966369X.2018.1428536.
60	Sovacool, B. K., L. Baker, M. Martiskainen, and A. Hook. 2019. Processes of elite power and low-carbon pathways: Experimentation, financialisation, and dispossession. *Global Environmental Change* 59: 101985. doi:10.1016/j.gloenvcha.2019.101985.
61	Ensor, J. E., S. E. Park, S. J. Attwood, A. M. Kaminski, and J. E. Johnson. 2018. Can community-based adaptation increase resilience? *Climate and Development* 10. Taylor & Francis: 134–151. doi:10.1080/17565529.2016.1223595.
62	Hirons, M., E. Boyd, C. McDermott, R. Asare, A. Morel, J. Mason, Y. Malhi, and K. Norris. 2018. Understanding climate resilience in Ghanaian cocoa communities – Advancing a biocultural perspective. *Journal of Rural Studies* 63: 120–129. doi:10.1016/j.jrurstud.2018.08.010.
63	Davidson, J. L., C. Jacobson, A. Lyth, A. Dedekorkut-Howes, C. L. Baldwin, J. C. Ellison, N. J. Holbrook, M. J. Howes, et al. 2016. Interrogating resilience: toward a typology to improve its operationalization. *Ecology and Society* 21. Resilience Alliance: 27. doi:10.5751/ES-08450-210227.
64	Haf, S., K. Parkhill, M. McDonald, and G. Griffiths. 2019. Distributing power? Community energy projects' experiences of planning, policy and incumbents in the devolved nations of Scotland and Wales. *Journal of Environmental Planning and Management* 62. Routledge: 921–938. doi:10.1080/09640568.2018.1453490.
65	Roddis, P., S. Carver, M. Dallimer, P. Norman, and G. Ziv. 2018. The role of community acceptance in planning outcomes for onshore wind and solar farms: An energy justice analysis. *Applied Energy* 226: 353–364. doi:10.1016/j.apenergy.2018.05.087.
66	Bee, B. A. 2016. Power, perception, and adaptation: Exploring gender and social–environmental risk perception in northern Guanajuato, Mexico. *Geoforum* 69: 71–80. doi:10.1016/j.geoforum.2015.12.006.
67	Rao, N., C. Singh, D. Solomon, L. Camfield, R. Sidiki, M. Angula, P. Poonacha, A. Sidibé, et al. 2020. Managing risk, changing aspirations and household dynamics: Implications for wellbeing and adaptation in semi-arid Africa and India. *World Development* 125: 104667. doi:10.1016/j.worlddev.2019.104667.
68	Sova, C., J. Vervoort, T. Thornton, A. Helfgott, D. Matthews, and A. Chaudhury. 2015. Exploring farmer preference shaping in international agricultural climate change

	adaptation regimes. *Environmental Science & Policy* 54: 463–474. doi:10.1016/j.envsci.2015.08.008.
69	Henrique, K. P., and P. Tschakert. 2019. Contested grounds: Adaptation to flooding and the politics of (in)visibility in São Paulo's eastern periphery. *Geoforum* 104: 181–192. doi:10.1016/j.geoforum.2019.04.026.
70	McKendry, C. 2016. Cities and the challenge of multiscalar climate justice: climate governance and social equity in Chicago, Birmingham, and Vancouver. *Local Environment* 21. Routledge: 1354–1371. doi:10.1080/13549839.2015.1116064.
71	Paterson, S. K., M. Pelling, L. H. Nunes, F. de Araújo Moreira, K. Guida, and J. A. Marengo. 2017. Size does matter: City scale and the asymmetries of climate change adaptation in three coastal towns. *Geoforum* 81: 109–119. doi:10.1016/j.geoforum.2017.02.014.
72	Lakhanpal, S. 2019. Contesting renewable energy in the global south: A case-study of local opposition to a wind power project in the Western Ghats of India. *Environmental Development* 30: 51–60. doi:10.1016/j.envdev.2019.02.002.
73	Singh, C. 2018. Is participatory watershed development building local adaptive capacity? Findings from a case study in Rajasthan, India. *Environmental Development* 25: 43–58. doi:10.1016/j.envdev.2017.11.004.
74	Gonda, N. 2019. Re-politicizing the gender and climate change debate: The potential of feminist political ecology to engage with power in action in adaptation policies and projects in Nicaragua. *Geoforum* 106: 87–96. doi:10.1016/j.geoforum.2019.07.020.
75	Nightingale, A. J. 2017. Power and politics in climate change adaptation efforts: Struggles over authority and recognition in the context of political instability. *Geoforum* 84: 11–20. doi:10.1016/j.geoforum.2017.05.011.
76	Allen, E. 2019. Women's leadership in renewable transformation, energy justice and energy democracy: Redistributing power. *Energy Research and Social Science* 57. Northeastern University. doi:10.1016/j.erss.2019.101233.
77	Daly, M., and L. Dilling. 2019. The politics of "usable" knowledge: examining the development of climate services in Tanzania. *Climatic Change* 157: 61–80. doi:10.1007/s10584-019-02510-w.
78	Holland, B. 2017. Procedural justice in local climate adaptation: political capabilities and transformational change. *Environmental Politics* 26. Lehigh University: 391–412. doi:10.1080/09644016.2017.1287625.
79	Karlsson, L., L. O. Naess, A. Nightingale, and J. Thompson. 2018. "Triple wins' or "triple faults'? Analysing the equity implications of policy discourses on climate-smart agriculture (CSA). *Journal of Peasant Studies* 45: 150–174. doi:10.1080/03066150.2017.1351433.

80	Taylor Aiken, G., L. Middlemiss, S. Sallu, and R. Hauxwell-Baldwin. 2017. Researching climate change and community in neoliberal contexts: an emerging critical approach. *WIREs Climate Change* 8: e463. doi:10.1002/wcc.463.
81	Woroniecki, S., R. Krüger, A. Rau, M. S. Preuss, N. Baumgartner, S. Raggers, L. Niessen, L. Holländer, et al. 2019. The framing of power in climate change adaptation research. *WIREs Climate Change* 10. doi:10.1002/wcc.617.
82	Dunlap, A. 2018. Counterinsurgency for wind energy: the Bíi Hioxo wind park in Juchitán, Mexico. *The Journal of Peasant Studies* 45. Routledge: 630–652. doi:10.1080/03066150.2016.1259221.
83	Hoang, C. 2019. 'This is my garden': justice claims and struggles over forests in Vietnam's REDD+. *Climate Policy* 19. UAB Instituto de Ciencia y Tecnología Ambientales; University of East Anglia; Vietnam Academy of Social Sciences: S23–S35. doi:10.1080/14693062.2018.1527202.
84	De Jong, M., H. Stout, and L. Sun. 2017. Seeing the People's Republic of China through the Eyes of Montesquieu: Why Sino-European Collaboration on Eco City Development Suffers from European Misinterpretations of "Good Governance." *Sustainability* 9. Multidisciplinary Digital Publishing Institute: 151. doi:10.3390/su9020151.
85	Djoudi, H., B. Locatelli, C. Vaast, K. Asher, M. Brockhaus, and B. Basnett Sijapati. 2016. Beyond dichotomies: Gender and intersecting inequalities in climate change studies. *Ambio* 45: 248–262. doi:10.1007/s13280-016-0825-2.
86	Eastin, J. 2018. Climate change and gender equality in developing states. *World Development* 107: 289–305. doi:10.1016/j.worlddev.2018.02.021.
87	Kammerbauer, M., and C. Wamsler. 2017. Social inequality and marginalization in post-disaster recovery: Challenging the consensus? *International Journal of Disaster Risk Reduction* 24: 411–418. doi:10.1016/j.ijdrr.2017.06.019.
88	Haverkamp, J. A. 2017. Politics, values, and reflexivity: The case of adaptation to climate change in Hampton Roads, Virginia. *Environment and Planning A: Economy and Space* 49. SAGE Publications Ltd: 2673–2692. doi:10.1177/0308518X17707525.
89	Wood, B. T., A. J. Dougill, C. H. Quinn, and L. C. Stringer. 2016. Exploring Power and Procedural Justice Within Climate Compatible Development Project Design: Whose Priorities Are Being Considered? *The Journal of Environment & Development* 25. SAGE Publications Inc: 363–395. doi:10.1177/1070496516664179.
90	Wood, B. T., A. J. Dougill, L. C. Stringer, and C. H. Quinn. 2018. Implementing Climate-Compatible Development in the Context of Power: Lessons for Encouraging Procedural Justice through Community-Based Projects. *Resources* 7. Multidisciplinary Digital Publishing Institute: 36. doi:10.3390/resources7020036.
91	Omukuti, J. 2020. Challenging the obsession with local level institutions in country ownership of climate change adaptation. *Land Use Policy* 94: 104525. doi:10.1016/j.landusepol.2020.104525.

92	Vij, S., R. Biesbroek, A. Groot, K. Termeer, and B. P. Parajuli. 2019. Power interplay between actors: using material and ideational resources to shape local adaptation plans of action (LAPAs) in Nepal. *Climate Policy* 19. Taylor & Francis: 571–584. doi:10.1080/14693062.2018.1534723.
93	Huber, A., and D. Joshi. 2015. Hydropower, Anti-Politics, and the Opening of New Political Spaces in the Eastern Himalayas. *World Development* 76: 13–25. doi:10.1016/j.worlddev.2015.06.006.
94	Singh, C., M. Tebboth, D. Spear, P. Ansah, and A. Mensah. 2019. Exploring methodological approaches to assess climate change vulnerability and adaptation: reflections from using life history approaches. *Regional Environmental Change* 19: 2667–2682. doi:10.1007/s10113-019-01562-z.
95	Bertana, A. 2020. The role of power in community participation: Relocation as climate change adaptation in Fiji. *Environment and Planning C: Politics and Space* 38. SAGE Publications Ltd STM: 902–919. doi:10.1177/2399654420909394.
96	Koslov, L. 2019. Avoiding Climate Change: “Agnostic Adaptation” and the Politics of Public Silence. *Annals of the American Association of Geographers* 109. Taylor & Francis: 568–580. doi:10.1080/24694452.2018.1549472.
97	Buggy, L., and K. E. McNamara. 2016. The need to reinterpret “community” for climate change adaptation: a case study of Pele Island, Vanuatu. *Climate and Development* 8: 270–280. doi:10.1080/17565529.2015.1041445.
98	Bremer, S., A. Blanchard, N. Mamnun, M. Stiller-Reeve, M. M. Haque, and E. Tvinnereim. 2017. Narrative as a Method for Eliciting Tacit Knowledge of Climate Variability in Bangladesh. *Weather, Climate, and Society* 9. American Meteorological Society: 669–686. doi:10.1175/WCAS-D-17-0007.1.
99	Geun Ji, H. 2019. The evolution of the policy environment for climate change migration in Bangladesh: Competing narratives, coalitions and power. *Development Policy Review* 37: 603–620. doi:10.1111/dpr.12384.
100	Ober, K., and P. Sakdapolrak. 2020. Whose climate change adaptation ‘barriers’? Exploring the coloniality of climate change adaptation policy assemblages in Thailand and beyond. *Singapore Journal of Tropical Geography* 41: 86–104. doi:https://doi.org/10.1111/sjtg.12309.
101	Ongolo, S., and A. Karsenty. 2015. The politics of forestland use in a cunning government: lessons for contemporary forest governance reforms. *CIFOR*. doi:10.1505/146554815815500561.
102	Prys, M., and T. Wojczewski. 2015. Rising Powers, NGOs and North-South Relations in Global Climate Governance: The Case of Climate Finance. *Politikon* 42: 93–111. doi:10.1080/02589346.2015.1005794.

103	Ciplet, D. 2017. Subverting the status quo? Climate debt, vulnerability and counter-hegemonic frame integration in United Nations climate politics – a framework for analysis. *Review of International Political Economy* 24. Routledge: 1052–1075. doi:10.1080/09692290.2017.1392336.
104	Scoville-Simonds, M., H. Jamali, and M. Hufty. 2020. The Hazards of Mainstreaming: Climate change adaptation politics in three dimensions. *World Development* 125: 104683. doi:10.1016/j.worlddev.2019.104683.
105	Di Gregorio, M., L. Fatorelli, J. Paavola, B. Locatelli, E. Pramova, D. R. Nurrochmat, P. H. May, M. Brockhaus, et al. 2019. Multi-level governance and power in climate change policy networks. *Global Environmental Change* 54: 64–77. doi:10.1016/j.gloenvcha.2018.10.003.
106	Sultana, P., and P. M. Thompson. 2017. Adaptation or conflict? Responses to climate change in water management in Bangladesh. *Environmental Science & Policy* 78: 149–156. doi:10.1016/j.envsci.2017.09.011.
107	Gillard, R., A. Gouldson, J. Paavola, and J. Van Alstine. 2016. Transformational responses to climate change: beyond a systems perspective of social change in mitigation and adaptation. *WIREs Climate Change* 7: 251–265. doi:10.1002/wcc.384.
108	Mcmanus, P. 2015. Geographic Connections: Language, Power Relations, and Relevance. *Geographical Research* 53: 349–356. doi:10.1111/1745-5871.12152.
109	Wilbanks, T. J. 2015. Putting “Place” in a multiscale context: Perspectives from the sustainability sciences. *Environmental Science & Policy* 53. Place as a Boundary Device for the Sustainability Sciences: 70–79. doi:10.1016/j.envsci.2015.04.009.
110	Lavorel, S., M. J. Colloff, B. Locatelli, R. Gorddard, S. M. Prober, M. Gabillet, C. Devaux, D. Laforgue, et al. 2019. Mustering the power of ecosystems for adaptation to climate change. *Environmental Science & Policy* 92: 87–97. doi:10.1016/j.envsci.2018.11.010.
111	Parkhill, K. A., F. Shirani, C. Butler, K. L. Henwood, C. Groves, and N. F. Pidgeon. 2015. ‘We are a community [but] that takes a certain amount of energy’: Exploring shared visions, social action, and resilience in place-based community-led energy initiatives. *Environmental Science & Policy* 53. Place as a Boundary Device for the Sustainability Sciences: 60–69. doi:10.1016/j.envsci.2015.05.014.
112	Ravera, F., V. Reyes-García, U. Pascual, A. G. Drucker, D. Tarrasón, and M. R. Bellon. 2019. Gendered agrobiodiversity management and adaptation to climate change: differentiated strategies in two marginal rural areas of India. *Agriculture and Human Values* 36: 455–474. doi:10.1007/s10460-018-09907-w.
113	Feldpausch-Parker, A. M., T. R. Peterson, J. C. Stephens, and E. J. Wilson. 2018. Smart grid electricity system planning and climate disruptions: A review of climate and energy discourse post-Superstorm Sandy. *Renewable and Sustainable Energy Reviews* 82: 1961–1968. doi:10.1016/j.rser.2017.06.015.

114	Morchain, D., D. Spear, G. Ziervogel, H. Masundire, M. N. Angula, J. Davies, C. Molefe, and S. Hegga. 2019. Building transformative capacity in southern Africa: Surfacing knowledge and challenging structures through participatory Vulnerability and Risk Assessments. *Action Research* 17. SAGE Publications: 19–41. doi:10.1177/1476750319829205.
115	Schroering, C. 2019. Water is a Human Right! Grassroots Resistance to Corporate Power. *Journal of World-Systems Research* 25: 28–34. doi:10.5195/jwsr.2019.899.
116	Abel, N., R. M. Wise, M. J. Colloff, B. H. Walker, J. R. A. Butler, P. Ryan, C. Norman, A. Langston, et al. 2016. Building resilient pathways to transformation when "no one is in charge": Insights from Australia's murray-darling basin. *Ecology and Society* 21. doi:10.5751/ES-08422-210223.
117	Bastakoti, R. R., and C. Davidsen. 2017. Framing REDD+ at National Level: Actors and Discourse around Nepal's Policy Debate. *Forests* 8. Multidisciplinary Digital Publishing Institute: 57. doi:10.3390/f8030057.
118	Sova, C. A., T. F. Thornton, R. Zougmore, A. Helfgott, and A. S. Chaudhury. 2017. Power and influence mapping in Ghana's agricultural adaptation policy regime. *Climate and Development* 9. Taylor & Francis: 399–414. doi:10.1080/17565529.2016.1154450.
119	Joshi, D., J. Platteeuw, J. Singh, and J. Teoh. 2019. Watered down? Civil society organizations and hydropower development in the Darjeeling and Sikkim regions, Eastern Himalaya: A comparative study. *Climate Policy* 19. Taylor & Francis: S63–S77. doi:10.1080/14693062.2018.1557035.
120	Jones, N. 2019. "It tastes like heaven": Critical and embodied food pedagogy with Black youth in the Anthropocene. *Policy Futures in Education* 17. SAGE Publications: 905–923. doi:10.1177/1478210318810614.
121	Thaler, T., and S. Seebauer. 2019. Bottom-up citizen initiatives in natural hazard management: Why they appear and what they can do? *Environmental Science & Policy* 94: 101–111. doi:10.1016/j.envsci.2018.12.012.
122	Jones, L., Ludi, E., Jeans, H., & Barihaihi, M. (2019) Revisiting the Local Adaptive Capacity framework: learning from the implementation of a research and programming framework in Africa. *Climate and Development*, 11(1), 3-13.
123	Wamsler, C., and S. Raggers. 2018. Principles for supporting city–citizen commoning for climate adaptation: From adaptation governance to sustainable transformation. *Environmental Science and Policy* 85: 81–89. Scopus. doi:10.1016/j.envsci.2018.03.021.
124	Sovacool, B. K., M. Tan-Mullins, and W. Abrahamse. 2018. Bloated bodies and broken bricks: Power, ecology, and inequality in the political economy of natural disaster recovery. *World Development* 110: 243–255. doi:10.1016/j.worlddev.2018.05.028.
125	Aldunce, P., R. Beilin, J. Handmer, and M. Howden. 2016. Stakeholder participation in building resilience to disasters in a changing climate. *Environmental Hazards* 15. Taylor & Francis: 58–73. doi:10.1080/17477891.2015.1134427.

126	Hölscher, K., N. Frantzeskaki, T. McPhearson, and D. Loorbach. 2019. Capacities for urban transformations governance and the case of New York City. *Cities* 94: 186–199. doi:10.1016/j.cities.2019.05.037.
127	Jeff Birchall, S. 2020. Coastal climate adaptation planning and evolutionary governance: Insights from Homer, Alaska. *Marine Policy* 112. doi:10.1016/j.marpol.2018.12.029.
128	Mosberg, M., E. Nyukuri, and L. O. Naess. 2017. The power of 'know-who': Adaptation to climate change in a changing humanitarian landscape in Isiolo, Kenya. *IDS Bulletin* 48: 79–92. Scopus. doi:10.19088/1968-2017.154.
129	Taylor, M. 2016. Risky Ventures: Financial Inclusion, Risk Management and the Uncertain Rise of Index-Based Insurance. In *Risking Capitalism*, 31:237–266. Research in Political Economy. Emerald Group Publishing Limited. doi:10.1108/S0161-723020160000031013.
130	Tanjeela, M., and S. Rutherford. 2018. The Influence of Gender Relations on Women's Involvement and Experience in Climate Change Adaptation Programs in Bangladesh. *SAGE Open* 8. SAGE Publications: 2158244018812620. doi:10.1177/2158244018812620.
131	Clissold, R., K. E. McNamara, and R. Westoby. 2020. Barriers to adaptation: Insights from Laamu Atoll, Maldives. *Asia Pacific Viewpoint* 61: 381–390. doi:10.1111/apv.12258.
132	De Wit, S. 2018. Victims or masters of adaptation? How the idea of adaptation to climate change travels up and down to a village in Simanjiro, Maasailand northern Tanzania. *Sociologus* 68: 21–41.
133	Van Huynh, C., C. T. van Scheltinga, T. H. Pham, N. Q. Duong, P. T. Tran, L. H. K. Nguyen, T. G. Pham, N. B. Nguyen, et al. 2019. Drought and conflicts at the local level: Establishing a water sharing mechanism for the summer-autumn rice production in Central Vietnam. *International Soil and Water Conservation Research* 7: 362–375. doi:10.1016/j.iswcr.2019.07.001.
134	Ben Nasr, J., and M. S. Bachta. 2018. Conflicts and water governance challenge in irrigated areas of semi-arid regions. *Arabian Journal of Geosciences* 11: 753. doi:10.1007/s12517-018-4075-4.
135	Sultana, P., Thompson, P. M., Paudel, N. S., Pariyar, M., & Rahman, M. (2019). Transforming local natural resource conflicts to cooperation in a changing climate: Bangladesh and Nepal lessons. *Climate Policy*, *19*(sup1), S94-S106.
136	Zulu, L. C., E. A. Adams, R. Chikowo, and S. Snapp. 2018. The role of community-based livestock management institutions in the adoption and scaling up of pigeon peas in Malawi. *Food Policy* 79: 141–155. doi:10.1016/j.foodpol.2018.06.007.
137	Anderson, C., and J. Schirmer. 2015. An Empirical Investigation of Social Capital and Networks at Local Scale through Resistance to Lower-Carbon Infrastructure. *Society & Natural Resources* 28. Routledge: 749–765. doi:10.1080/08941920.2015.1014599.

138	Mavhura, E. 2017. Building resilience to food insecurity in rural communities: Evidence from traditional institutions in Zimbabwe. *Jàmbá : Journal of Disaster Risk Studies* 9: 453. doi:10.4102/jamba.v9i1.453.
139	Muchacha, M., and M. Mushunje. 2019. The gender dynamics of climate change on rural women's agro-based livelihoods and food security in rural Zimbabwe: implications for green social work. *Critical and Radical Social Work* 7: 59–72. doi:10.1332/204986019X15491042559655.
140	Guerbois, C., U. Brady, A. G. de Swardt, and C. Fabricius. 2019. Nurturing ecosystem-based adaptations in South Africa's Garden Route: a common pool resource governance perspective. *Regional Environmental Change* 19: 1849–1863. doi:10.1007/s10113-019-01508-5.
141	Aubin, D., C. Riche, V. Vande Water, and I. La Jeunesse. 2019. The adaptive capacity of local water basin authorities to climate change: The Thau lagoon basin in France. *Science of The Total Environment* 651: 2013–2023. doi:10.1016/j.scitotenv.2018.10.078.
142	Scott, D., K. N. Iipinge, J. K. E. Mfune, D. Muchadenyika, O. V. Makuti, and G. Ziervogel. 2018. The story of water in windhoek: A narrative approach to interpreting a transdisciplinary process. *Water (Switzerland)* 10. Scopus. doi:10.3390/w10101366.
143	Taylor, M. 2018. Climate-smart agriculture: what is it good for? *The Journal of Peasant Studies* 45. Routledge: 89–107. doi:10.1080/03066150.2017.1312355.
144	Udas, P. B., D. D. Tamang, A. Unni, M. Hamal, K. Shrestha, and A. Pandit. 2019. Basin level gendered vulnerabilities and adaptation: A case of Gandaki River Basin. *Environmental Development* 31. Conceptualizing and Contextualizing Gendered Vulnerabilities to Climate Variability in the Hindu Kush Himalayan Region: 43–54. doi:10.1016/j.envdev.2019.05.002.
145	Senini, E. 2018. Farm Saved Seeds: A Right to Use or a Right Abused? *European Food and Feed Law Review* 13. Lexxion Verlagsgesellschaft mbH: 116–124.
146	Dunlap, A. 2018. Insurrection for land, sea and dignity: resistance and autonomy against wind energy in Álvaro Obregón, Mexico. *Journal of Political Ecology* 25. University of Arizona Libraries. doi:10.2458/v25i1.22863.
147	Fraser, A. 2017. The missing politics of urban vulnerability: The state and the co-production of climate risk. *Environment and Planning A: Economy and Space* 49. SAGE Publications Ltd: 2835–2852. doi:10.1177/0308518X17732341.
148	Poudyal, M., B. S. Ramamonjisoa, N. Hockley, O. S. Rakotonarivo, J. M. Gibbons, R. Mandimbiniaina, A. Rasoamanana, and J. P. G. Jones. 2016. Can REDD+ social safeguards reach the 'right' people? Lessons from Madagascar. *Global Environmental Change* 37: 31–42. doi:10.1016/j.gloenvcha.2016.01.004.

149	Uson, M. A. M. 2017. Natural disasters and land grabs: the politics of their intersection in the Philippines following super typhoon Haiyan. *Canadian Journal of Development Studies / Revue canadienne d'études du développement* 38. Routledge: 414–430. doi:10.1080/02255189.2017.1308316.
150	MacArthur, J., and S. Matthewman. 2018. Populist resistance and alternative transitions: Indigenous ownership of energy infrastructure in Aotearoa New Zealand. *Energy Research & Social Science* 43. Sustainable Energy Transformations in an Age of Populism, Post-Truth Politics, and Local Resistance: 16–24. doi:10.1016/j.erss.2018.05.009.
151	Temper, L. 2019. Blocking pipelines, unsettling environmental justice: from rights of nature to responsibility to territory. *Local Environment* 24: 94–112. doi:10.1080/13549839.2018.1536698.
152	Hackfort, S., and H.-J. Burchardt. 2018. Analyzing socio-ecological transformations – a relational approach to gender and climate adaptation. *Critical Policy Studies* 12: 169–186. doi:10.1080/19460171.2016.1191363.
153	Osborne, N. 2015. Intersectionality and kyriarchy: A framework for approaching power and social justice in planning and climate change adaptation. *Planning Theory* 14: 130–151. doi:10.1177/1473095213516443.
154	Sova, C. A., A. Helfgott, A. S. Chaudhury, D. Matthews, T. F. Thornton, and S. J. Vermeulen. 2015. Multi-level Stakeholder Influence Mapping: Visualizing Power Relations Across Actor Levels in Nepal's Agricultural Climate Change Adaptation Regime. *Systemic Practice and Action Research* 28: 383–409. Scopus. doi:10.1007/s11213-014-9335-y.
155	Wamsler, C., J. Alkan-Olsson, H. Björn, H. Falck, H. Hanson, T. Oskarsson, E. Simonsson, and F. Zelmerlow. 2020. Beyond participation: when citizen engagement leads to undesirable outcomes for nature-based solutions and climate change adaptation. *Climatic Change* 158: 235–254. doi:10.1007/s10584-019-02557-9.
156	Lebel, L., M. Käkönen, V. Dany, P. Lebel, T. Thuon, and S. Voladet. 2018. The framing and governance of climate change adaptation projects in Lao PDR and Cambodia. *International Environmental Agreements: Politics, Law and Economics* 18: 429–446. Doi:10.1007/s10784-018-9397-x
157	Woroniecki, S. 2019. Enabling Environments? Examining Social Co-Benefits of Ecosystem-Based Adaptation to Climate Change in Sri Lanka. *Sustainability* 11. Multidisciplinary Digital Publishing Institute: 772. doi:10.3390/su11030772.
158	Boer, H. J. 2019. Deliberative engagement and REDD+ in Indonesia. *Geoforum* 104: 170–180. doi:10.1016/j.geoforum.2019.04.025.
159	Godfrey-Wood, R. 2016. Political Challenges of Addressing Climate Change through the 'Entrepreneurial State.' *IDS Bulletin* 47. doi:10.19088/1968-2016.187.
160	Ravera, F., B. Martín-López, U. Pascual, and A. Drucker. 2016. The diversity of gendered adaptation strategies to climate change of Indian farmers: A feminist intersectional approach. *Ambio* 45: 335–351. doi:10.1007/s13280-016-0833-2.

161	Sayer, J., C. Margules, A. K. Boedhihartono, A. Dale, T. Sunderland, J. Supriatna, and R. Saryanthi. 2015. Landscape approaches; what are the pre-conditions for success? *Sustainability Science* 10: 345–355. doi:10.1007/s11625-014-0281-5.
162	Woroniecki, S., C. Wamsler, and E. Boyd. 2019. The promises and pitfalls of ecosystem-based adaptation to climate change as a vehicle for social empowerment. *Ecology and Society* 24. The Resilience Alliance. doi:10.5751/ES-10854-240204.
163	Blok, A. 2016. Assembling urban riskscapes: Climate adaptation, scales of change and the politics of expertise in Surat, India. *City* 20. Routledge: 602–618. doi:10.1080/13604813.2016.1194000.
164	Jordan, J. C. 2019. Deconstructing resilience: why gender and power matter in responding to climate stress in Bangladesh. *Climate and Development* 11: 167–179. doi:10.1080/17565529.2018.1442790.
165	Chu, E. K. 2018. Transnational Support for Urban Climate Adaptation: Emerging Forms of Agency and Dependency. *Global Environmental Politics* 18: 25–46. doi:10.1162/glep_a_00467.
166	Renner, T., and S. Meijerink. 2018. Policy entrepreneurs in international river basins—getting climate adaptation on the cross-border water policy agenda. *Regional Environmental Change* 18: 1287–1298. doi:10.1007/s10113-017-1178-5.
167	Boezeman, D. 2016. Understanding the transformation of climate futures. A conceptual framework illustrated with urban adaptation policy. *Futures* 76. Policy-Making for the Long Term: Puzzling and Powering to Navigate Wicked Futures Issues: 30–41. doi:10.1016/j.futures.2015.07.008.
168	van der Steen, M., N. Chin-A-Fat, M. Vink, and M. van Twist. 2016. Puzzling, powering and perpetuating: Long-term decision-making by the Dutch Delta Committee. *Futures* 76. Policy-Making for the Long Term: Puzzling and Powering to Navigate Wicked Futures Issues: 7–17. doi:10.1016/j.futures.2016.01.001.
169	Lo, K., and V. Castán Broto. 2019. Co-benefits, contradictions, and multi-level governance of low-carbon experimentation: Leveraging solar energy for sustainable development in China. *Global Environmental Change* 59: 101993. doi:10.1016/j.gloenvcha.2019.101993.
170	Bell, F. M. 2019. Collective survival strategies and anti-colonial practice in ecosocial work. *Journal of Community Practice* 27. University of Michigan, Ann Arbor; University of Manitoba; Loyola University of Chicago: 279–295. doi:10.1080/10705422.2019.1652947.
171	Destradi, S., and C. Jakobeit. 2015. Global Governance Debates and Dilemmas: Emerging Powers' Perspectives and Roles in Global Trade and Climate Governance. *Strategic Analysis* 39. Routledge: 60–72. doi:10.1080/09700161.2014.980538.

172	Givens, J. E., J. Padowski, C. D. Guzman, K. Malek, R. Witinok-Huber, B. Cosens, M. Briscoe, J. Boll, et al. 2018. Incorporating Social System Dynamics in the Columbia River Basin: Food-Energy-Water Resilience and Sustainability Modeling in the Yakima River Basin. *Frontiers in Environmental Science* 6. Frontiers. doi:10.3389/fenvs.2018.00104.
173	Kremen, C., and A. M. Merenlender. 2018. Landscapes that work for biodiversity and people. *Science* 362. American Association for the Advancement of Science: eaau6020. doi:10.1126/science.aau6020.
174	Batel, S. 2018. A critical discussion of research on the social acceptance of renewable energy generation and associated infrastructures and an agenda for the future. *Journal of Environmental Policy & Planning* 20. Routledge: 356–369. doi:10.1080/1523908X.2017.1417120.
175	Sareen, S., and K. Rommetveit. 2019. Smart gridlock? Challenging hegemonic framings of mitigation solutions and scalability. *Environmental Research Letters* 14. IOP Publishing: 075004. doi:10.1088/1748-9326/ab21e6.
176	Garriga-López, A. 2019. Puerto Rico: The Future In Question. *Shima: The International Journal of Research into Island Cultures* 13. doi:10.21463/shima.13.2.13.
177	Trell, E.-M., and M. T. van Geet. 2019. The Governance of Local Urban Climate Adaptation: Towards Participation, Collaboration and Shared Responsibilities. *Planning Theory & Practice* 20. Routledge: 376–394. doi:10.1080/14649357.2019.1629573.
178	Hamilton, M. 2018. Understanding what shapes varying perceptions of the procedural fairness of transboundary environmental decision-making processes. *Ecology and Society* 23. Resilience Alliance Inc.
179	Brugnach, M., M. Craps, and A. Dewulf. 2017. Including indigenous peoples in climate change mitigation: addressing issues of scale, knowledge and power. *Climatic Change* 140: 19–32. doi:10.1007/s10584-014-1280-3.
180	Steiner, C. 2015. A Sea of Warriors: Performing an Identity of Resilience and Empowerment in the Face of Climate Change in the Pacific. *The Contemporary Pacific* 27: 147–180. doi:10.1353/cp.2015.0002.
181	Khoza, S., D. Van Niekerk, and L. D. Nemakonde. 2019. Understanding gender dimensions of climate-smart agriculture adoption in disaster-prone smallholder farming communities in Malawi and Zambia. *Disaster Prevention and Management: An International Journal* 28. Emerald Publishing Limited: 530–547. doi:10.1108/DPM-10-2018-0347.
182	Lindström, A., and A. Ruud. 2017. Whose Hydropower? From Conflictual Management into an Era of Reconciling Environmental Concerns; a Retake of Hydropower Governance towards Win-Win Solutions? *Sustainability* 9. Multidisciplinary Digital Publishing Institute: 1262. doi:10.3390/su9071262.
183	Ratter, B., A. Hennig, and Zahid. 2019. Challenges for shared responsibility – Political and social framing of coastal protection transformation in the Maldives. *DIE ERDE – Journal of the Geographical Society of Berlin* 150: 169–183. doi:10.12854/erde-2019-426.

184	Yadav, B. D., H. R. Bigsby, and I. MacDonald. 2016. Elitism: normative ethics of local organisation in community-based natural resources management. *International Journal of Organizational Analysis* 24. Emerald Group Publishing Limited: 932–955. doi:10.1108/IJOA-06-2015-0873.
185	Westholm, L., and S. Arora-Jonsson. 2018. What room for politics and change in global climate governance? Addressing gender in co-benefits and safeguards. *Environmental Politics* 27. Routledge: 917–938. doi:10.1080/09644016.2018.1479115.
186	Neef, A., L. Benge, B. Boruff, N. Pauli, E. Weber, and R. Varea. 2018. Climate adaptation strategies in Fiji: The role of social norms and cultural values. *World Development* 107: 125–137. doi:10.1016/j.worlddev.2018.02.029.
187	Lindegaard, L. S. 2018. Adaptation as a political arena: Interrogating sedentarization as climate change adaptation in Central Vietnam. *Global Environmental Change* 49: 166–174. doi:10.1016/j.gloenvcha.2018.02.012.

Coding process

The collected articles underwent a rigorous two-phase coding process. First, deductive coding systematically categorised articles into 38 predefined themes across six coding clusters (see Table S3). Next, inductive thematic analysis facilitated deeper exploration, revealing five key thematic areas: (1) decision-making processes, (2) negotiation, contestation, and conflict, (3) participation and shared learning, (4) elements contributing to success, and (5) obstacles to meaningful participation. Within each theme, inductive coding was employed to identify approximately ten specific sub-themes, allowing a nuanced examination of how participation operates in diverse contexts, from top-down, government-led processes to bottom-up, community-driven initiatives.

Table S3: Coding clusters for the complete set of journal articles reviewed

**Key clusters for coding of dataset**	Specific sub-themes (coding categories)
**Significance across climate-related areas**	Short summary of article; article type; adaptation; mitigation; resilience; social transformation; extreme weather events and climate-related disasters; water, food and livelihood security; peri-urban and urban; rural, agricultural and agrarian; other climate-related areas.
**Actors and location**	Actors and stakeholders; axes of inequality; geographical area; within the same spatial scale; across-scale interactions.
**Power**	Invisible power; hidden power; visible power; interactions and

	entanglements of power; injustices and struggles.
**Politics and processes of negotiation**	Values, interests, experiences and rights of different actor groups or stakeholders; subject(s) of resilience making; processes of decision-making; deliberation, contestation, and negotiation; sharing learning and participation; compromises and trade-offs; features of success; obstacles to success; sustaining processes
**Methodology**	Methodology employed to collect data; data analysis approaches; replicability of methods; novelty of methodology.
**Other**	Quality of article; additional notes; article hyperlink.

The coding process incorporated binomial, categorical, and text-based coding methods. Binomial coding was applied to variables that could be classified in a yes/no format, such as whether an article explicitly discussed participation in climate adaptation, mitigation, or resilience efforts. By adopting such a process, we were able to ensure that we systematically captured which articles addressed key dimensions of participatory methods. Categorical coding was employed to categorise articles into predefined thematic categories. For instance, we categorised articles based on the geographical focus (Global North vs. Global South), type of participatory approach (top-down, consultative, co-productive, transformative), and the primary stakeholder groups involved (e.g., governments, NGOs, Indigenous communities, private sector, local grassroots organisations). Categorical coding was particularly useful in identifying trends in participation across governance levels and institutional settings. Text-based coding also offered us a more nuanced qualitative analysis, which helped us capture how participation was framed and experienced within different climate initiatives. It also allowed us to code direct quotes, themes, discursive framings of participation, and specific case study descriptions. Text-based coding was fundamental in the second coding stage (inductive thematic analysis), enabling a more in-depth analysis of how power was distributed and negotiated within participatory processes. By employing a mixed-methods coding approach, we ensured that our review captured nuances in participatory approaches within climate mitigation, adaptation and resilience-building efforts.

A key analytical step was identifying patterns of exclusion and marginalisation within participatory climate action. For example, stakeholder groups were categorised based on how they were included or excluded from decision-making processes, whether through tokenistic consultation, structural barriers, or elite-driven processes. We used colour coding

to organise findings and trace connections across different scales to visualise the relationships between participation and power. By coding across multiple clusters— such as participation, power, and negotiation— the research team could investigate how issues related to race, gender, disability, and other intersecting inequalities were addressed (or not) within the different types of participation described in the papers.

The final stage of the review involved synthesising findings to examine how participation operates across different contexts, highlighting trends and key barriers to transformative participation. The final three participation categories—Domination & Exclusion, Negotiation & Contestation, and Transformation—were developed based on this systematic coding process. By employing this structured but iterative methodology, our systematic review offers a critical assessment of participatory approaches deployed within climate mitigation and adaptation, shedding light on the constraints, opportunities, and transformative potential of participation in climate mitigation, adaptation, and resilience-building efforts.

A critical aspect of the analysis was the geographical distribution of studies. The majority of studies (68%) focused on Global South contexts, while 32% examined Global North cases. However, most author teams were based in the Global North, reinforcing persistent epistemic biases in participatory climate research. Top-down, exclusionary processes often characterise Global South participatory processes, while Global North cases emphasise deliberative participatory approaches that remain constrained by government or expert control. To account for feedback loops between different participation stages, we also analysed how participatory approaches can reinforce existing power hierarchies or enable shifts toward more inclusive models. Through this structured but iterative methodology, the systematic review critically assesses participatory approaches in climate mitigation, adaptation, and resilience-building efforts, illuminating both their constraints and transformative potential.
